# Highly Stable Aqueous Zinc Metal Batteries Enabled by an Ultrathin Crack‐Free Hydrophobic Layer with Rigid Sub‐Nanochannels

**DOI:** 10.1002/advs.202303773

**Published:** 2023-07-28

**Authors:** Dongming Xu, Xueting Ren, Yan Xu, Yijiang Wang, Shibin Zhang, Benqiang Chen, Zhi Chang, Anqiang Pan, Haoshen Zhou

**Affiliations:** ^1^ School of Materials Science and Engineering Key Laboratory of Electronic Packaging and Advanced Functional Materials of Hunan Province Central South University Changsha Hunan 410083 P. R. China; ^2^ Center of Energy Storage Materials and Technology College of Engineering and Applied Sciences Jiangsu Key Laboratory of Artificial Functional Materials National Laboratory of Solid State Micro‐structures and Collaborative Innovation Center of Advanced Micro‐structures Nanjing University Nanjing 210093 P. R. China

**Keywords:** aqueous zinc metal batteries, dendrite‐free Zn, de‐solvation, metal–organic frameworks, Zn anodes

## Abstract

Aqueous zinc‐metal batteries (AZMBs) have received tremendous attentions due to their high safety, low cost, environmental friendliness, and simple process. However, zinc‐metal still suffer from uncontrollable dendrite growth and surface parasitic reactions that reduce the Coulombic efficiency (CE) and lifetime of AZMBs. These problems which are closely related to the active water are not well‐solved. Here, an ultrathin crack‐free metal–organic framework (ZIF‐7*_x_*‐8) with rigid sub‐nanopore (0.3 nm) is constructed on Zn‐metal to promote the de‐solvation of zinc‐ions before approaching Zn‐metal surface, reduce the contacting opportunity between water and Zn, and consequently eliminate water‐induced corrosion and side‐reactions. Due to the presence of rigid and ordered sub‐nanochannels, Zn‐ions deposits on Zn‐metal follow a highly ordered manner, resulting in a dendrite‐free Zn‐metal with negligible by‐products, which significantly improve the reversibility and lifespan of Zn‐metals. As a result, Zn‐metal protected by ultrathin crack‐free ZIF‐7*_x_*‐8 layer exhibits excellent cycling stability (over 2200 h) and extremely‐high 99.96% CE during 6000 cycles. The aqueous PANI‐V_2_O_5_//ZIF‐7*_x_*‐8@Zn full‐cell preserves 86% high‐capacity retention even after ultra‐long 2000 cycles. The practical pouch‐cell can also be cycled for more than 120 cycles. It is believed that the simple strategy demonstrated in this work can accelerate the practical utilizations of AZMBs.

## Graphical abstract

An ultrathin crack‐free hydrophobic metal–organic framework layer (ZIF‐7*_x_*‐8) with rigid sub‐nanopores (0.3 nm) is constructed on Zn surface to promote the de‐solvation of zinc‐ions before they approach Zn‐metal surface, thus reducing the contacting opportunity between water and Zn‐metal, and consequently eliminating water‐induced corrosion and side reactions. In addition, Zn‐ions tend to experience a highly ordered deposition on Zn metal surface.

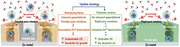

## 1 Introduction

**Figure 1:**
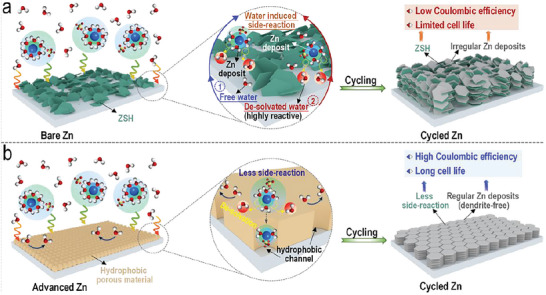
a) Exposed Zn foil under aqueous ZnSO_4_ electrolyte. H_2_O causing severe water passivation and dendrite formation, and leading to aqueous zinc‐metal batteries with low coulombic efficiency and limited lifespan. b) The importance of constructing hydrophobic porous material with narrow pore windows in promoting the de‐solvation of Zn‐ions, reducing water induced side‐reactions, and improving the coulombic efficiency and lifespan of aqueous zinc‐metal batteries (AZMBs).

Aqueous zinc metal batteries (AZMBs) have attracted tremendous research attention due to their inherent safety, low cost, and high ionic conductivity, as well as the suitable standard electrode potential of the Zn metal anode (−0.76 V, vs standard hydrogen electrode) and high theoretical capacity (5855 mAh cm^−3^ or 820 mAh g^−1^).^[^ ^1^, ^2^ ^]^ The high energy density of zinc metal batteries is influenced by the electrodes, the electrolytes, and the manufacturing process.^[^ ^3^, ^4^, ^5^ ^]^ Unfortunately, Zn metal anodes face several critical problems that hinder their further practical applications. For example, the inherent thermodynamic instability of metallic Zn in aqueous electrolytes would lead to inevitable parasitic reactions including corrosion and hydrogen precipitation at the electrode/electrolyte interface.^[^ ^6^, ^7^ ^]^ Typically, during cycling, before approaching the Zn metal surface, solvated Zn‐ions tend to experience de‐solvation process and release parts of electrochemically reactive water molecules. The presence of free‐water combined with highly reactive de‐solvated water further corrode Zn metal and leads to the accumulation of by‐products (Zn_4_(OH)_6_SO_4_·*x*H_2_O, shorted as ZHS) which would reduce the CE and lifespan of Zn metals.^[^ ^8^, ^9^, ^10^ ^]^ Even worse, during the plating/stripping process, the uncontrolled growth of Zn dendrites on Zn metal can further damage the reversibility and working time of Zn metals, thus resulting in fast cell failure (**Figure** **1**a). The occurrence of these problems further leads to poor cycle life, capacity degradation, low CE, and in severe cases, cell short circuiting.^[^ ^11^, ^12^ ^]^

The interface between metallic Zn and the aqueous electrolyte essentially determines the behavior of Zn^2+^ ions being plated and stripped from the electrode. These inherent detrimental problems such as hydrogen precipitation and parasitic reactions at the interface of Zn metals is closely related to the presence of reactive water.^[^ ^13^, ^14^ ^]^ Current efforts to stabilize the Zn metal interface mainly focused on designing and modulating of the crystal plane, preparing alloy anodes, optimizing electrolytes structures, and constructing various artificial solid electrolyte interfaces.^[^ ^15^, ^16^, ^17^, ^18^, ^19^, ^20^, ^21^, ^22^, ^23^ ^]^ Admitting those mentioned strategies can inhibit the growth of Zn dendrites and suppress side reactions to some extent, they however, fail to simultaneously regulate orderly deposition of zinc ions while reduce the contact probability between Zn metal and reactive water molecules). Very recently, researchers began to coat porous materials with nano scale/sub‐nano scale channels on Zn metal surface as protective layers to reduce the contact possibility between Zn metal and reactive water molecules, thus mitigating Zn metal free from water corrosion.^[^ ^24^ ^]^ The ordered channels of those porous materials can also guide the uniform deposition of Zn‐ions in an orderly manner and promote Zn metal with dendrite‐free surfaces, thus enable Zn metal with high coulombic efficiency and long cycle life.^[^ ^25^, ^26^, ^27^, ^28^ ^]^ Yet, those reported strategies also have several drawbacks that counteracted the positive effects brought by porous materials coated on Zn metal surface. For example, for most works, porous materials were mixed with binders before directly coated on Zn metal surfaces.^[^ ^29^, ^30^ ^]^ Generally, from a macro perspective, the thick coating layer would additionally increase cell polarization, thus slow down the Zn‐ion plating/stripping rate and decrease the Zn‐ion transfer efficiency. Moreover, since most of the porous materials were synthesized in advance before mixing with polymeric binders and coating on Zn surface, various inherent defects (from the porous material themselves) and gaps between particles can be found on the coating layers. Those imperfect sites would offset parts of the positive effects induced by porous material coating layer since free water molecules and solvated Zn‐ions would penetrate through those gaps and directly contact metallic Zn.^[^ ^31^, ^32^ ^]^ In addition, from a micro perspective, for most of the porous materials especially MOFs, their pore windows were flexible and usually experienced size changing, which may allow larger solvated ion clusters to pass through the telescopically vibrating pore during ion migration, thus leading to incomplete de‐solvation toward solvated Zn‐ions.^[^ ^33^, ^34^ ^]^ These afore‐mentioned problems can prevent the carefully designed sub‐nanopore channels from fully realizing their unique functions, and incomplete de‐solvation can diminish the positive effect of the MOF artificial interfacial layer.^[^ ^35^ ^]^ Constructing an ultra‐thin and crack‐free porous material layer with rigid hydrophobic narrow pore windows on Zn metal surface is expected to overcome those afore‐mentioned drawbacks and hold promising prospects in suppressing water induced side‐reactions and promoting ordered Zn‐ion deposition. Consequently, AZMBs with high CE and long lifespan can be obtained (Figure 1b). However, it is still highly challenging to find proper porous material which can satisfy all of those above‐mentioned characteristics.

In this work, an ultrathin crack‐free metal–organic framework layer (ZIF‐7*_x_*‐8) with hydrophobic rigid 0.3 nm sub‐nano pores was in situ grown on Zn metal surface by a fast current‐driven synthesis (FCDS) method within minutes. The hydrophobic crack‐free mixed‐linker ZIF‐7*_x_*‐8 layer with rigid pore window further enhanced the de‐solvation of solvated Zn‐ions and effectively suppressed the contact between water molecules and metallic Zn, thus effectively suppressing water‐induced side reactions. Meanwhile, the ordered and rigid sub‐nano channels of ZIF‐7*_x_*‐8 redistributed Zn‐ions at the electrode/electrolyte interface and inhibited dendrite formation by regulating uniform Zn‐ion flux. As a result, symmetrical cell based on a Zn metal protected by ultrathin crack‐free ZIF‐7*_x_*‐8 could be cycled stably for as long as 2200 h (at a current density of 1.0 mA cm^−2^ and a deposition capacity of 1.0 mAh cm^−2^). Even when tested under much harsh condition (10 mA cm^−2^, 2 mA cm^−2^), the corresponding symmetrical cell still delivered more than 1100 h lifespan. The assembled Zn//Cu half‐cells achieved high CEs of 99.72%, 99.91%, and 99.96% at 1 mA cm^−2^/1 mAh cm^−2^, 5 mA cm^−2^/1 mAh cm^−2^, and 10 mA cm^−2^/1 mAh cm^−2^, respectively. PANI‐V_2_O_5_ (PVO)//ZIF‐7*_x_*‐8@Zn full‐cell delivered a high specific capacity of 202.9 mAh g^−1^ at a current density of 5 A g^−1^ and preserved 86% of its original capacity after ultra‐long 2000 cycles. Pouch‐cell assembled with NH_4_V_4_O_10_ (NVO) and ZIF‐7*_x_*‐8@Zn exhibited a high capacity of 190.4 mAh g^−1^ at a current density of 0.553 A g^−1^ and maintained about 78.83% its capacity after 120 cycles.

## 2 Results and Discussions

**Figure 2:**
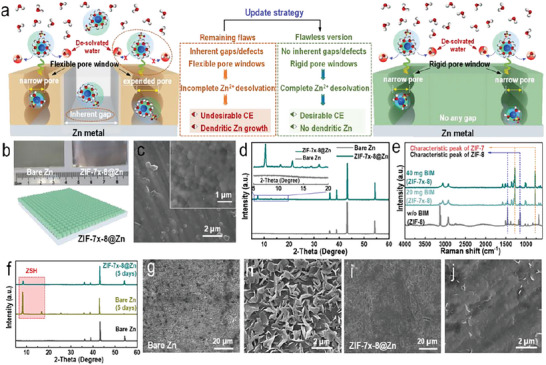
a) The importance of constructing ultrathin crack‐free with rigid pore windows in promoting the de‐solvation of Zn‐ions. b) Digital photographs of bare Zn and ZIF‐7*_x_*‐8@Zn. c) SEM images of the ZIF‐7*_x_*‐8@Zn. d) XRD patterns of bare Zn and ZIF‐7*_x_*‐8@Zn. e) Raman spectra of the ZIF‐7*_x_*‐8@Zn with different amount of benzimidazole (BIM) additions. f) XRD patterns of bare Zn and ZIF‐7*_x_*‐8@Zn metal before and after being soaked in aqueous 2 m ZnSO_4_ electrolyte for 5 days. SEM image of g,h) bare Zn and i,j) ZIF‐7*_x_*‐8@Zn after being soaked in aqueous 2 m ZnSO_4_ for 5 days.

To fulfill the role of de‐solvating the Zn‐ions, the pore windows of porous material used should be smaller than that of the size of hydrated Zn‐ions. As one typical porous material which possesses sub‐nano channels of about 0.34 nm, ZIF‐8 was frequently employed in various situations. It provides abundant space for ion migration due to its homogeneous pores and abundant porosity.^[^ ^33^, ^36^, ^37^ ^]^ We also would like to use it to protect the Zn metal since its channels are slightly smaller than the size of Zn‐ions. Yet, as have been discussed before, the pore windows of ZIF‐8 are flexible and usually experienced size changing, which may diminish the positive effect of the ZIF‐8 as it may possibly lead to incomplete de‐solvation toward Zn‐ions. It was reported that exposing and or preparing ZIF‐8 under electric field can induce the lattice distortion of ZIF‐8, thus stiffening the pore windows and eliminating pore flexibility.^[^ ^38^, ^39^ ^]^ Replacing parts of the 2‐methylimidazole (2‐MIM) group within ZIF‐8 with benzimidazole (BIM) can further narrow and reinforce the pore windows, which we thought was beneficial in further improving de‐solvation toward Zn‐ions. Based on the afore‐mentioned discussion, a special FCDS method and mixed‐linker linkage strategy was used to in situ construct ultrathin crack‐free MOF layer (ZIF‐7*_x_*‐8) with hydrophobic rigid pore window of 0.3 nm on metallic Zn surface. We expected Zn metal in situ grown with ultrathin crack‐free and hydrophobic ZIF‐7*_x_*‐8 layer to overcome those afore‐mentioned drawbacks and hold promising prospects in suppressing water induced side‐reactions, and promoting ordered Zn‐ion deposition, thus further improving the CE and lifespan of AZMBs (**Figure** **2**a). As schematically illustrated in Figure S1, Supporting Information, when Zn foil and graphite rod was employed as electrodes and immersed into a mixed solution of 2‐MIM, BIM and a suitable amount of zinc acetate dihydrate (Zn(CH_3_COO)_2_), a direct current of 0.5 mA cm^−2^ was utilized to promote the deprotonation of ligands 2‐MIM and BIM. The Zn^2+^ ions could be attracted and bound to the deprotonated ligands, and resulting in a continuous co‐growth membranes layer on the surface of Zn foil. The in situ electric field permitted the formation of ZIF‐8 as the parent skeleton, while the addition of BIM molecules further narrows and reinforces the pore size by partially replacing parts of the initial 2‐MIM linkers (Figure S2, Supporting Information). The formation of insulating ZIF layer prevented further contact between the mother liquor and the Zn foil. After the Zn foil surface was fully covered by insulating ZIF‐7*_x_*‐8 layer, the voltage increased quickly, then the current‐driven film growth stoped automatically (Figure S3, Supporting Information). This self‐inhibited growth property was utilized to achieve monolayer ZIF. The appearance of optical interference phenomenon on the Zn foil surface indicated that the ZIF layer had a dense and smooth surface at the submicron scale (Figure 2b and Figure S4, Supporting Information). Scanning electron microscopy (SEM) images showed the continuous crack‐free ZIF‐7*_x_*‐8 membranes adhered well to the Zn foil substrate (Figure 2c and Figure S5, Supporting Information). Both the XRD and Raman results of ZIF‐7*_x_*‐8@Zn samples obtained with different BIM additions demonstrated distinct characteristic peaks of ZIF‐7 and ZIF‐8 (Figure 2d,e). The FTIR spectra (Figure S6, Supporting Information) of ZIF‐7*_x_*‐8@Zn showed the signals of BIM and MIM. The peak located at 742 and 1469 cm^−1^ could be ascribed to the δC─H out‐of‐plane bending vibration and the δ_as_C═C asymmetric stretching vibration of BIM, respectively. These results strongly indicated the successful introduction of BIM linker into ZIF‐8, in other words the successful formation of ZIF‐7*_x_*‐8 on Zn foil surface.

In order to further analyze the corrosion resistance of Zn anode protected by ZIF‐7*_x_*‐8 mixed‐linker membranes, both ZIF‐7*_x_*‐8@Zn and bare Zn were immersed into aqueous 2 m ZnSO_4_ electrolyte for 5 days at the same time. XRD results showed that after immersion, the intensity of the characteristic peak of zinc hydroxide sulfate (Zn_4_SO_4_(OH)_6_·4H_2_O, shorted as ZHS) on ZIF‐7*_x_*‐8@Zn was very weak and much less than the super strong characteristic peak of byproducts on bare Zn (Figure 2f). After 5 days of immersion, various by‐products were covered on the surface of the bare Zn foil (Figure 2g,h), suggesting that the bare Zn was severely corroded. In contrast, only tiny by‐products appeared on the surface of ZIF‐7*_x_*‐8@Zn. This could be due to the excellent anti‐corrosion ability of the prepared ZIF‐7*_x_*‐8 (Figure 2i,j and Figure S7, Supporting Information). The contact angle of 2 m ZnSO_4_·H_2_O on the ZIF‐7*_x_*‐8‐Zn surface was 139.2°, which was much larger than that on the bare Zn surface (94.2°), indicating that the ZIF‐7*_x_*‐8 mixed‐linker membranes were hydrophobic and thus could alleviate the water‐induced chemical corrosion of the Zn foil (Figure S8a,b, Supporting Information). Compared with that of the bare Zn, ZIF‐7*_x_*‐8@Zn demonstrated not only much lower corrosion current and positively shifted corrosion potential, but also greatly reduced the hydrogen‐evolution reaction (Figure S8c,d, Supporting Information). The bare Zn and ZIF‐7*_x_*‐8@Zn after 5 days’ immersion (sample from Figure 2f–i) was then used to fabricate symmetrical cells. Symmetric cell based on bare Zn after 5 days’ immersion showed drastic voltage fluctuations after only 50 h, in which a very fast cell short circuit occurred. In contrast, symmetric cell based on ZIF‐7*_x_*‐8@Zn after 5 days’ immersion cycled stably for 261 h before failure (Figure S9a, Supporting Information). The corresponding XRD results further suggested that the in situ formed crack‐free ZIF‐7*_x_*‐8 layer was effective in suppressing water‐related side‐reaction products even after being soaked for 5 days and re‐circulation (Figure S9b, Supporting Information). The SEM images of the cycled bare Zn after 5 days’ immersion showed an uneven surface which was full of severe Zn dendrites (Figure S10a–d, Supporting Information). In contrast, the surface of the ZIF‐7*_x_*‐8@Zn after cycling was flat and smooth, and covered by densely packed deposited Zn without visible Zn dendrites (Figure S10e–h, Supporting Information). These above discussed evidences indicated the good ability of the ZIF‐7*_x_*‐8 interphase in suppressing water induced side reactions and corrosion of Zn metal.

At a constant potential, the relationship between current and time can sensitively reflect the nucleation process and surface behavior of the Zn metal. Therefore, we performed chronoamperometry (CA) tests on a symmetric cell based on both bare Zn and ZIF‐7*_x_*‐8@Zn with an applied overpotential of 150 mV (Figure S11a, Supporting Information). Clearly, result of cell based on ZIF‐7*_x_*‐8@Zn demonstrated that Zn^2+^ started a stable 3D diffusion after a brief 2D diffusion, which finally resulting in a dense Zn nucleation. In stark contrast, the current density based on bare Zn continuously increased and exhibited a planar diffusion process with random nucleation. The absorbed Zn^2+^ ions tended to diffuse laterally along the bare Zn surface to find the most favorable energy location for charge transfer to aggregate and trigger dendrite formation. We suspected that due to the existence of this ultrathin crack‐free and hydrophobic ZIF‐7*_x_*‐8 layer which possesses 0.3 nm pore windows, before reaching Zn metal, solvated Zn^2+^ were expected to shed‐off parts of the water molecules within their solvation sheaths as they pass through the channels during electrochemical deposition. This design could effectively suppress the contact opportunities between various highly reactive water molecules and Zn metal, thus reducing the possibility of Zn corrosion. And, the highly porous channel structure could homogenize the Zn^2+^ flux and guided its deposition between the protective layer and the Zn metal surface. In addition, the resistivity of the ZIF‐7*_x_*‐8@Zn‐based symmetric cell was lower than that of the bare Zn‐based symmetric cell, indicating excellent ionic conductivity (Figure S11b, Supporting Information).

**Figure 3:**
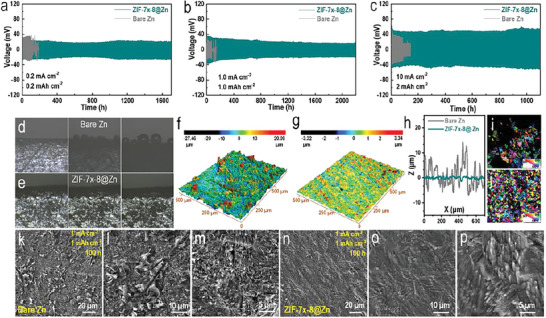
a–c) Time‐voltage profiles for symmetrical cells based on both bare Zn and ZIF‐7*_x_*‐8@Zn under different current and capacity conditions (0.2 mAh cm^−2^ at 0.2 mA cm^−2^, 1 mAh cm^2^ at 1 mA cm^−2^, and 10 mAh cm^−2^ at 2 mA cm^−2^, respectively). In situ optical microscopy images of symmetric transparent cell based on d) bare Zn and e) ZIF‐7*_x_*‐8@Zn at a current density of 10 mA cm^−1^. The Reconstructed 3D topography of the Laser Scanning Confocal Microscopy (LSCM) of f) cycled bare Zn and g) cycled ZIF‐7*_x_*‐8@Zn after 100 h under 1 mAh cm^−2^ at 1 mA cm^−2^. h) The surface height difference of the bare Zn and ZIF‐7*_x_*‐8@Zn after cycling. The electron backscatter diffraction (EBSD) images of i) cycled bare Zn and j) cycled ZIF‐7*_x_*‐8@Zn. SEM images of k–m) cycled bare Zn metals and h–p) cycled ZIF‐7*_x_*‐8@ Zn metals under condition of 1 mAh cm^−2^ at 1 mA cm^−2^.

To verify our conjecture, symmetric cells based both bare Zn and ZIF‐7*_x_*‐8@Zn were assembled and tested. When measured under condition of 0.2 mAh cm^−2^ at 0.2 mA cm^−2^ f, Zn metal covered with ultrathin crack‐free and hydrophobic ZIF‐7*_x_*‐8 layer exhibited an ultra‐long lifetime of more than 1700 h, which was much better than that of cell based on bare Zn (showed fluctuating voltage profile and went short‐circuit rapidly after only 160 h) (**Figure** **3**a). When tested under 1.0 mAh cm^−2^ at 1.0 mA cm^−2^, cell assembled with ZIF‐7*_x_*‐8@Zn survived for more than 2200 h, which was about 20 times longer than that of the cell based on bare Zn (Figure 3b). Similar improvements were observed for the symmetric cells based on ZIF‐7*_x_*‐8@Zn tested under other conditions (Figure S12, Supporting Information). More surprisingly, even when tested under harsh current condition of 10.0 mA cm^−2^ (with capacity of 2 or 3.3 mAh cm^−2^), the cycle life of symmetric cell based on ZIF‐7*_x_*‐8@Zn was still much longer than that of the symmetric cell assembled with bare Zn (Figure 3c). The improvement could also be achieved when measured under aqueous electrolytes based different zinc‐salts including Zn(CF_3_SO_3_)_2_ and ZnClO_4_ (Figure S13, Supporting Information). The flat and smooth dendrite‐free surface of the ZIF‐7*_x_*‐8@Zn also proved this conclusion (Figures S14 and S15, Supporting Information). An in situ optical microscopy test was performed to further observe the morphology changes of bare Zn and ZIF‐7*_x_*‐8@Zn. The photographed images illustrated that hydrogen precipitation occurred on bare Zn surface at a current density of 10.0 mA cm^−2^ within only 10 min, and became much severer with increasing time (Figure 3d). In sharp contrast, with the increase of deposition time, the black area of ZIF‐7*_x_*‐8@Zn anode became wider when measured under the same conditions, corresponding to the gradual deposition of zinc. And, no hydrogen precipitation reaction was observed (Figure 3e). This directly verified the fact that the ultra‐thin crack‐free and hydrophobic ZIF‐7*_x_*‐8 layer was beneficial in protecting Zn metal from being corroded through preventing reactive water molecules from reaching Zn surface. Confocal laser scanning microscopy of the two kinds of cycled Zn electrodes were performed (Figure 3f–h). Clearly, for bare Zn after cycling 100 h under condition of 1.0 mAh cm^−2^ at 1.0 mA cm^−2^, its rough surface was covered by obvious by‐products/Zn dendrites and the produced dendrites were entangled with glass fibers. The surface contour lines of the cycled bare Zn exhibited a 20 µm height difference which suggested occurrence of serious side‐reactions and dendritic Zn growth (Figure 3f,h and Figure S16a,b, Supporting Information). In sharp contrast, the ZIF‐7*_x_*‐8@Zn under the same condition however, demonstrated a much flatter surface and uniform color in the 2D images (Figure 3g and Figure S16c,d, Supporting Information). The corresponding height difference was merely about 2 µm, indicating significantly suppressed side‐reactions and dendritic Zn growth. SEM images of cycled Zn metals at a current density of 1.0 mA cm^−2^ and capacity of 1.0 mAh cm^−2^ also reflected the same results (Figure 3k–p). In order to further verify the effect of ZIF‐7*_x_*‐8 layer in regulating the deposition behavior of Zn‐ions, the surface orientation analysis of bare Zn and ZIF‐7*_x_*‐8@Zn after cycling was studied by using electron backscatter diffraction (EBSD) technique (Figure 3i,j; Figure S17 and Table S1, Supporting Information). The cycled bare Zn exhibited irregular signal and low acquisition rate of 24%, which indicated high roughness of Zn. This could be ascribed to the random aggregation of Zn deposits and even severe corrosion in some areas. In stark contrast, the acquisition rate was up to more than 50% on the ZIF‐7*_x_*‐8@Zn anode, suggesting much regular and ordered Zn deposition.

**Figure 4:**
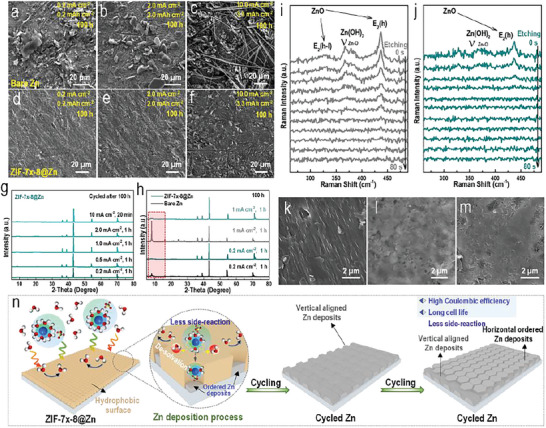
The effectiveness of ZIF‐7*_x_*‐8 mix‐liker membranes in suppressing the water related by‐products and formation of dendritic Zn. SEM images of a–c) cycled bare Zn metals and d–f) cycled ZIF‐7*_x_*‐8@Zn metals under different current and capacity conditions (0.2 mAh cm^−2^ at 0.2 mA cm^−2^, 2.0 mAh cm^−2^ at 2.0 mA cm^−2^, and 3.3 mAh cm^−2^ at 10 mA cm^−2^, respectively). g–h) XRD patterns of bare Zn and ZIF‐7*_x_*‐8@Zn metal anodes after cycled for 100 h under different current densities. i,j) Etching Raman spectroscopy of cycled bare Zn and cycled ZIF‐7*_x_*‐8@Zn. k–m) SEM images of cycled ZIF‐7*_x_*‐8@Zn metals under different cycles (150th, 200th, and 300th, respectively). n) Schematic illustration of the importance effectiveness of ultrathin crack‐free ZIF‐7*_x_*‐8 mix‐liker membranes in suppressing the water related by‐products and formation of dendritic Zn.

The morphologies of both Zn and ZIF‐7*_x_*‐8@Zn cycled under different current densities and deposition capacities (0.2 mAh cm^−2^ at 0.2 mA cm^−2^, 0.5 mAh cm^−2^ at 0.5 mA cm^−2^, and 3.3 mAh cm^−2^ at 10 mA cm^−2^) were also recorded after 100 h (**Figure** **4**a–c and Figure S18, Supporting Information). After cycling for 100 h, the surface of bare Zn without ZIF‐7*_x_*‐8 layer was sparse with numerous protrusions and nanosheet‐like products (ZHS and Zn deposits). The ZHS and Zn dendrites produced on bare Zn surface under high current density conditions pierced the glass fibers and entangled with the fibers, consequently leading to cell short‐circuit. In sharp contrast, the surface of the cycled ZIF‐7*_x_*‐8@Zn electrodes were flat and smooth with no obvious dendrites or ZHS could be found (Figure 4d–f and Figure S19, Supporting Information). To further study the effectiveness of the ultra‐thin crack‐free and hydrophobic ZIF‐7*_x_*‐8, Zn metal with half of its surface covered with ultra‐thin crack‐free and hydrophobic ZIF‐7*_x_*‐8 layer was prepared and assembled into symmetrical cell (as schematically illustrated in Figure S20a, Supporting Information). After cycled for 200 h, the ZIF‐7*_x_*‐8 protected section was deposited flat and uniform with regular and ordered Zn deposits (Figure S20b–d, Supporting Information), while the un‐protected section was rough and covered with pits (Figure S20g–f, Supporting Information). The XRD results of Zn, ZIF‐7*_x_*‐8@Zn electrodes after cycling for 100 h under different conditions were also recorded (Figure 4g,h and Figure S21, Supporting Information). Obviously, nearly none side‐reaction related byproducts could be found on all the ZIF‐7_x_‐8@Zn electrodes after cycling at different conditions. In stark contrast, cycled bare Zn metals demonstrated strong by‐products related characteristic peaks. The corresponding X‐ray photoelectron spectroscopy spectra collected on the surfaces of cycled ZIF‐7*_x_*‐8@Zn after 100 h did not exhibit apparent element S related peak, which further indicated the significance of ZIF‐7*_x_*‐8 in suppressing water‐induced by‐products (Figure S22, Supporting Information). To further verify the positive effect brought by the ZIF‐7*_x_*‐8 mixed‐linker layers on the Zn anodes, etching Raman of both cycled bare Zn and ZIF‐7*_x_*‐8@Zn was collected. Obviously, for the cycled bare Zn metal, strong peaks located at 437 and 329 cm^−1^ corresponding to ZnO and Zn(OH)_2_ can be observed even after 80 s etching (Figure 4i). On the contrary, only tiny peaks related to ZnO and Zn(OH)_2_ could be detected in the cycled ZIF‐7*_x_*‐8@Zn electrode, which indicated the positive effect of the ultrathin crack‐free and hydrophobic ZIF‐7*_x_*‐8 layer in promoting Zn deposits with preferred perpendicular direction but the thin thickness of the by‐products (Figure 4j).^[^ ^40^ ^]^ The SEM images of the symmetric cells based on ZIF‐7*_x_*‐8@Zn at different cycles (100th, 200th, and 300th) was tested (Figure 4k–m; Figures S23 and S24, Supporting Information). It could be seen from the SEM images that Zn^2+^ was first deposited on the surface of the Zn foil in a vertical form, which may be due to the effect of induced uniform ion flow in the pore channel. As the cumulative deposition increases, Zn^2+^ started to be deposited on the surface of the Zn foil in a horizontal direction. The homogenized electric field formed within the rigid narrow channels promoted the uniform deposition of ordered Zn^2+^, thus inhibiting the formation of Zn dendrites (Figure 4n).

**Figure 5:**
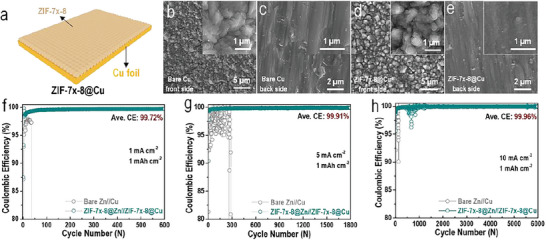
a) Schematic illustration and b,c) SEM images of bare Cu foil and d,e) ZIF‐7*_x_*‐8@Cu. f–h) Electrochemical performances of Zn//Cu half‐cells based on both bare Zn and bare Cu, and ZIF‐7*_x_*‐8@Zn and ZIF‐7*_x_*‐8@Cu under different current and capacity conditions (1 mAh cm^−2^ at 1 mA cm^−2^, 1 mAh cm^−2^ at 5 mA cm^−2^, and 1 mAh cm^−2^ at 10 mA cm^−2^, respectively).

To verify the positive effect of ZIF‐7*_x_*‐8 artificial interfacial layer on Zn ion deposition/dissolution efficiency, ultrathin crack‐free and hydrophobic ZIF‐7*_x_*‐8 heterogeneous membranes were deposited on Cu foil surface using the same method (**Figure** **5**a). Unlike the smooth Cu foil, an ultrathin layer of elliptical ZIF‐7*_x_*‐8 could be clearly observed on the Cu after fast electrodynamic deposition (Figure 5b–f and Figure S25, Supporting Information). The excellent CEs and cycling stability of the ZIF‐7*_x_*‐8@Cu//ZIF‐7*_x_*‐8@Zn cell indicated that ZIF‐7*_x_*‐8 mixed‐linker layers have efficient protection and suppression of dendrite formation on the Zn metal anode. The assembled ZIF‐7*_x_*‐8@Zn//ZIF‐7*_x_*‐8@Cu half‐cells could achieve an average CE of up to 99.7% even at lower current densities and deposition capacities (1.0 mA cm^−2^, 1.0 mAh cm^−2^), while bare Cu//Zn cells had poor CE and exhibit significant perturbations and failure after 100 plating/stripping cycles under the same conditions (Figure 5f). The charge/discharge voltage distributions of the two half‐cells at different cycles were shown in Figure S26a–d, Supporting Information. The initial voltage hysteresis of ZIF‐7*_x_*‐8@Zn//ZIF‐7*_x_*‐8@Cu cell at a current density of 1.0 mA cm^−2^ deposited for 1 h was about 38.1 mV. The average CE was 99.91% achieved under condition of 1.0 mAh cm^−2^ at 5.0 mA cm^−2^, and could be plating/stripping stably for 1800 times (Figure 5g). Its initial voltage hysteresis was about 64.1 mV, which was lower than that of 79.0 mV for bare Cu//Zn. At a current density of 10.0 mA cm^−2^ and a deposition capacity of 1.0 mAh cm^−2^, ZIF‐7*_x_*‐8@Zn//ZIF‐7*_x_*‐8@Cu could be stably plating/stripping up to 6000 times (1200 h) and delivered an extremely high average CE of 99.96% (Figure 5h). Similar trends could be observed when cells were tested under different conditions (Figures S26e,f and S27, Supporting Information).

**Figure 6:**
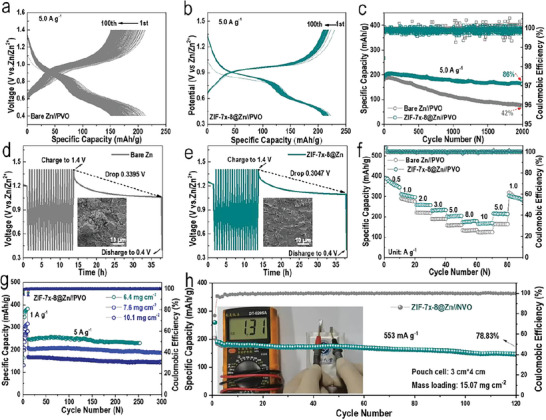
Electrochemical performances of aqueous zinc‐metal batteries (AZMBs) based on ZIF‐7*_x_*‐8@Zn. Voltage profile of cell based on a) PANI‐V_2_O_5_//bare Zn (PVO//bare Zn), and b) PVO//ZIF‐7*_x_*‐8@Zn at current density of 5.0 A g^−1^ and the corresponding. c) Long‐term cycling performances of the two cells. Self‐discharge of the PVO//Zn cell based on d) bare Zn and e) ZIF‐7*_x_*‐8@Zn at 5 A g^−1^ after 24 h rest. The insets were the corresponding SEM images of the bare Zn and ZIF‐7*_x_*‐8@Zn anode harvested from PVO//Zn cells at 5 A g^−1^ after 1000 cycles. f) Rate performance of NH_4_V_4_O_10_//Zn (NVO//Zn) half‐cells based on both bare Zn and ZIF‐7*_x_*‐8@Zn. g) Cycling performance of PVO//Zn half‐cells based on ZIF‐7*_x_*‐8@Zn metal under different cathode mass loadings. h) Cycling performance of PVO//Zn pouch‐cell based on ZIF‐7*_x_*‐8@Zn metal (the inset was the digital photo of pouch‐cell).

To examine the effect of ultrathin crack‐free and hydrophobic ZIF‐7*_x_*‐8 layers, full cells were assembled in a 2 m ZnSO_4_ electrolyte with PANI‐V_2_O_5_ as the cathode (Figure S28, Supporting Information). The results of cyclic voltammetric curves (CV) indicated that the voltage polarization of the ZIF‐7*_x_*‐8@Zn//PVO battery was lower than that of the cell based on bare Zn (Figure S29, Supporting Information). Compared to bare Zn, the PVO//Zn cell based on ZIF‐7*_x_*‐8@Zn also had better cycling performance and voltage profile even at a low current density of 100 mA g^−1^ (Figure S30, Supporting Information). **Figure** **6**a–c showed the charge/discharge curves and the corresponding long cycle performance of bare Zn//PVO and ZIF‐7*_x_*‐8@Zn//PVO at a current density of 5.0 A g^−1^, respectively. The PVO//ZIF‐7*_x_*‐8@Zn cell could released a specific capacity of 202.9 mAh g^−1^ with a CE of 100%, and still maintained a capacity retention of 86% even after 2000 long cycles, which was much higher than that of the cell based on bare Zn anode (42%). The cycling and specific capacity test results of multiple PVO//ZIF‐7*_x_*‐8‐Zn cells at the same current were generally consistent (Figure S31a, Supporting Information). Compared to the PVO//bare Zn cell, the PVO//ZIF‐7*_x_*‐8 cell showed significantly lower charge transfer resistance values (Figure S31b, Supporting Information). The effect of ZIF‐7*_x_*‐8 on self‐discharge in PVO//Zn battery was evaluated by measuring the voltage reduction of a fully charged battery after 20 h resting. As shown in the Figure 6d,e, the voltage reduction of ZIF‐7*_x_*‐8@Zn//PVO was 0.3047 V, while the bare Zn//PVO cell decreased by 0.3395 V, indicating that the ultrathin crack‐free and hydrophobic ZIF‐7*_x_*‐8 layer effectively mitigated the self‐discharge behavior. The cycling performance at different current densities was further investigated, and the specific capacities of the batteries assembled with bare Zn, ZIF‐7*_x_*‐8@Zn were almost the same at a current density of 0.5 mA cm^−2^, while ZIF‐7*_x_*‐8@Zn//PVO maintained a higher capacity than bare Zn//PVO at gradually increasing current densities (Figure 6f). The ZIF‐7*_x_*‐8@Zn surface maintained a uniform, rounded‐edge plating after more than 500 cycles. More interestingly, after cycled for 1000 times, the ZIF‐7*_x_*‐8@Zn preserved a flat and dendrite‐free surface with negligible surface by‐products, while the bare Zn anode had a rough and distinctly dendritic surface (inset in Figure 6e,f and Figure S32, Supporting Information). The cycling performance was tested separately for different PVO mass loads at a current density of 5 A g^−1^ to examine the stability of ZIF‐7*_x_*‐8@Zn. As shown in Figure 6g, the specific capacity of the ZIF‐7*_x_*‐8@Zn//PVO cells decayed with increasing PVO mass loading. And, the cell remains stable for 300 cycles with a capacity retention rate of nearly 100% even at a high load of 10.1 mg cm^−2^ (Figure S33, Supporting Information). The electrochemical performance of the Zn//K_0.27_MnO_2_ (K_0.27_MnO_2_ shorted as KMO) cell based on ZIF‐7*_x_*‐8@Zn anode was also significantly improved (Figure S34, Supporting Information). Compared with the bare Zn//KMO cell, ZIF‐7*_x_*‐8@Zn//KMO cell demonstrated much stable cycling stability. To meet the application requirements and evaluate the practicability of ZIF‐7*_x_*‐8@Zn anode, a size of 3 × 4 cm^2^ pouch cell with areal capacity ratio of negative to positive electrodes (N/P ratio) of 4.2 was assembled (mass loading of PVO: 15.07 mg cm^−2^). Its open‐circuit voltage was measured to be 1.31 V (inset in Figure 6h). The pouch cell delivered a high specific capacity of 190.4 mAh g^−1^ at a current density of 0.553 A g^−1^ and preserved 78.83% capacity retention after 120 cycles (Figure 6h and Figure S35, Supporting Information).

## 3 Conclusion

In summary, by in situ growing an ultrathin crack‐free metal–organic framework layer (ZIF‐7*_x_*‐8) with rigid sub‐nanopore (0.3 nm) on the surface of metallic Zn, both the Zn‐ion plating/stripping reversibility and life span of aqueous zinc‐metal batteries were significantly improved. Specifically, the hydrophobic ZIF‐7*_x_*‐8 with rigid 0.3 nm pore windows formed on Zn surface acted as a physical barrier to promote the de‐solvation of Zn‐ions and to suppress the contact opportunity between the metallic Zn and reactive water molecules, thus reducing the probability of Zn corrosion. Moreover, the designed pore structure further acted as a flux distributor to induce homogeneous Zn‐ions flux, and facilitated uniform and ordered deposition of Zn‐ions, which consequently led to a dendrite‐free Zn with negligible by‐products. As a result, Zn metal protected by ultrathin crack‐free ZIF‐7*_x_*‐8 layer exhibited excellent cycling stability (over 2200 h) and an extremely‐high coulombic efficiency of 99.96% during 6000 cycles. The aqueous PVO//ZIF‐7*_x_*‐8@Zn full‐cell preserved a high‐capacity retention of 86% even after ultra‐long 2000 cycles. More inspiringly, a practical PVO//ZIF‐7*_x_*‐8@Zn pouch‐cell was also assembled and cycled for more than 120 cycles with good stability. This work further solved two long‐lasting fatal but tricky problems in AZMBs and provided a promising option for practical application of AZMBs.

## Conflict of Interest

The authors declare no conflict of interest.

## Supporting information

Supporting Information

## Data Availability

The data that support the findings of this study are available from the corresponding author upon reasonable request.
