# Supplementary material for: Highly Stable Aqueous Zinc Metal Batteries Enabled by an Ultrathin Crack‐Free Hydrophobic Layer with Rigid Sub‐Nanochannels
Source: Adv Sci (Weinh). 2023 Jul 28;10(27):2303773. doi: 10.1002/advs.202303773 (PMC10520658; doi:10.1002/advs.202303773)
Supplement: Supplementary file 1 — Supporting Information [file ADVS-10-2303773-s001.pdf]

ADVANCED SCIENCE
Open Access

Supporting Information

for *Adv. Sci.*, DOI 10.1002/advs.202303773

Highly Stable Aqueous Zinc Metal Batteries Enabled by an Ultrathin Crack-Free Hydrophobic Layer with Rigid Sub-Nanochannels

Dongming Xu, Xueting Ren, Yan Xu, Yijiang Wang, Shibin Zhang, Benqiang Chen, Zhi Chang, Anqiang Pan* and Haoshen Zhou**

Supporting Information

Highly Stable Aqueous Zinc Metal Batteries Enabled by an Ultrathin Crack-free Hydrophobic Layer with Rigid Sub-nanochannels

Dongming Xu, Xueting Ren, Yan Xu, Yijiang Wang, Shibin Zhang, Benqiang Chen, Zhi Chang, Anqiang Pan*, Haoshen Zhou**

D. M. Xu, X.T. Ren, Y. Xu, Y. J. Wang, S. B. Zhang, B. Q. Chen, Prof. Z. Chang, Prof. A. Q. Pan

School of Materials Science and Engineering, Key Laboratory of Electronic Packaging and Advanced Functional Materials of Hunan Province, Central South University, Changsha, 410083, Hunan, China.

E-mail: zhichang@csu.edu.cn (Prof. Z. Chang), pananqiang@csu.edu.cn (Prof. A. Q. Pan)

Prof. H. S. Zhou
Center of Energy Storage Materials & Technology, College of Engineering and Applied Sciences, Jiangsu Key Laboratory of Artificial Functional Materials, National Laboratory of Solid State Micro-structures, and Collaborative Innovation Center of Advanced Micro-structures, Nanjing University, Nanjing, 210093, P. R. China.

E-mail: hszhou@nju.edu.cn (Prof. H. Zhou).

Keywords: Aqueous zinc metal battery, metal-organic framework, Zn anode, de-solvation

Experimental Section

1. Material Preparation

1.1 Preparation of ZIF-7_x-8@Zn anode: First, the Zn foil (100 μm or 20 μm) was cut to 3 cm × 4 cm before use and then ultrasonically cleaned in water and acetone for 30 min to remove the residues on the Zn surface. One side of the Zn foil was protected with tape and the

other side was used as a support for the electrically driven growth of the mixed-linker membranes. Zn acetate (0.22 g) and 2-MIM (0.16 g) were dissolved in methanol (20 mL), noted as solution A. Suitable BIM (20 mg, 40 mg, 60 mg) were dissolved in 10 mL of N, N-dimethylformamide (DMF). Recorded as solution B. The solutions of A and B were treated with ultrasonication to dissolve all the chemicals. Mix solution A and solution B.

An ultrathin crack-free metal-organic framework layer (ZIF-7_x-8) on the surface of Zn foil was prepared by fast current-driven synthesis (FCDS). The Zn foil was immersed in the prepared mixed solution and ZIF-7_x-8 was gradually grown on the Zn surface at a current density of 0.5 mA cm^{-2}. After 20 minutes of electrical driving, the Zn foil was soaked in ethanol overnight and washed three times with deionized water and ethanol. Then it was dried in a vacuum oven at 100 °C for 6 hours to obtain ZIF-7_x-8@Zn.

1.2 Preparation of PANI-V_2O_5(PVO): V_2O_5 powder (2 mmol) was dissolved in deionized water (60 mL). Concentrated hydrochloric acid was added to the above solution under continuous stirring to adjust the pH of the solution to 1-2. Aniline (400 µL) was then added to obtain a dark brown solution. The above solution was transferred to a 100 ml reactor and placed at 180°C for 20 hours. After cooling to room temperature, the dark colored product was washed three times with deionized water and ethanol. Finally, the above samples were dried overnight in an oven at 60°C.

1.3 Synthesis of $K_{0.27}MnO_2$: First, $KMnO_4$ (0.6 g) and D(+)-glucose (1.0 g) were completely dissolved in different amounts of deionized water (10 and 4 mL), respectively. After adding the former to the latter and stirring for 40 seconds, a brown gel was formed. The gels were then placed in an oven at 100 °C for 12 hours to obtain dry gels. The dry gel was then calcined at 400 °C for 2 hours in a muffle furnace at a heating rate of 10 °C min^{-1}. The final product was obtained after washing and drying with deionized water.

1.4 Electrolyte preparation: Zinc sulfate ($ZnSO_4·7H_2O$, Shanghai Aladdin Biochemical Technology Co., Ltd., >99%) was dissolved into the deionized water to obtain the 2 M $ZnSO_4$ electrolyte for Zn symmetric cells and pouch cells.

2. Electrochemical measurements:

A three-electrode system was used to measure the Tafel diagram at a sweep rate of 10 mV s^{-1} with Zn plate as the working electrode, Pt foil as the counter electrode and Ag/AgCl as the reference electrode, scanning in the voltage interval of -0.7 ~ -1.25 V. The hydrogen precipitation reaction potentials were recorded by linear scanning voltammetry at a scanning rate of 1 mV s^{-1} in a 2 M $ZnSO_4$ electrolyte. In the CR2032 type cell, glass fiber (GF/D) as the

diaphragm and 2 M $ZnSO_4$ as the electrolyte were assembled for electrochemical testing. Two 100 μm bare Zn or ZIF-7_x-8@Zn were used as electrodes to assemble the symmetrical cell, respectively. The Zn//Cu half-cells were assembled with Zn plates as the anode and 20 μm Cu foil as the cathode. PVO, carbon black and polyvinylidene fluoride (PVDF) were thoroughly mixed in n -methyl-2-pyrrolidone (NMP) solvent in a mass ratio of 7:2:1 and stirred for 2 hours to obtain a homogeneous viscous slurry. Then the mixture was applied to a stainless-steel mesh and dried in a vacuum oven at 80°C for 12 hours. The loading mass of the PVO electrode was approximately 2-3 mg cm^{-2}. The Zn//PVO battery was prepared with PVO electrode as cathode, bare Zn, ZIF-7_x-8@Zn as anodes and 2.0 M aqueous $ZnSO_4$ solution as electrolyte. The Zn-NVO pouch cell was assembled by encapsulating a ZIF-7_x-8@Zn (3.1 cm × 4.2 cm, 20 μm), a piece of glass fiber (3.5 cm × 4.5 cm), a NVO cathode (3 cm × 4 cm, 15-20 mg cm^{-2}) and 1.0 mL of $ZnSO_4$ electrolyte in an aluminium-plastic bag.

3. Morphology and Structure Characterization

Crystal structures were determined using a Bruker D8 Advance X-ray diffraction (XRD) diffractometer with Cu-Kα X-rays (λ =1.5406Å) radiation at a scan rate of 5 °/min. Scanning electron microscope (SEM) images were examined using Hitachi Regulus 8100. Contact angle measurements were carried out using a Lauda Scientific LSA100 contact angle meter. Raman measurements were performed with a confocal Raman spectrometer (Thermo Fischer DXR). Optical images of the in-situ growth of Zn dendrites were obtained on an optical microscope using a home-made in situ optical electrochemical cell. FTIR spectroscopy was performed on a Nicolet iS 10 infrared microscope spectrometers. Raman spectroscopy was performed by a LabRam HR Evolution microscope spectrometer. The reconstructed 3D topographies of cycled metal electrodes were characterized with the laser scanning confocal microscopy (LSCM, KEYENCE VHX-1000E). X-ray Photoelectron Spectroscopy (XPS) of cycled bare Zn and ZIF-7x-8@Zn was performed using a Thermo ESCALAB 250XI spectrometers. The distribution of crystalline surfaces of bare Zn, cycled bare Zn and cycled ZIF-7x-8@Zn were characterized by electron backscatter diffraction (EBSD, SEM-EDS-EBSD (Hitachi Regulus8100) equipped with a Nordly max3 EBSD detector).

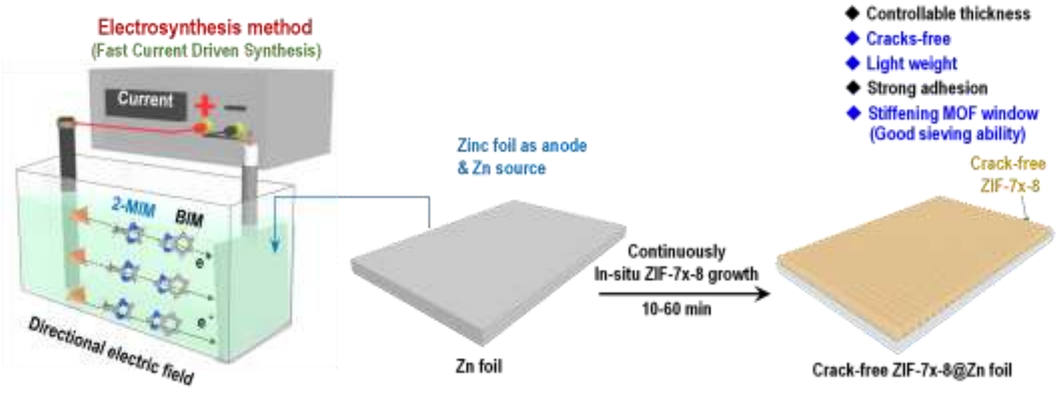

Figure S1. Schematic illustration of mixed-liker ZIF-7_x-8 membranes prepared by fast current-driven synthesis (FCDS) on Zn foil.

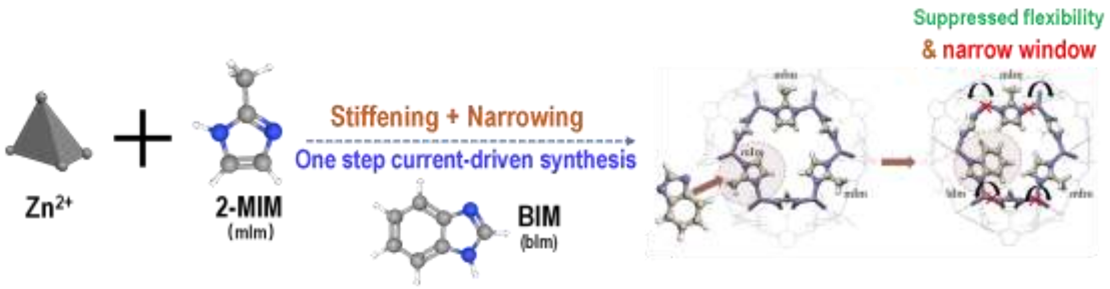

Figure S2. Structure illustration of ZIF-7_x-8 after BIM is connected to ZIF-8.

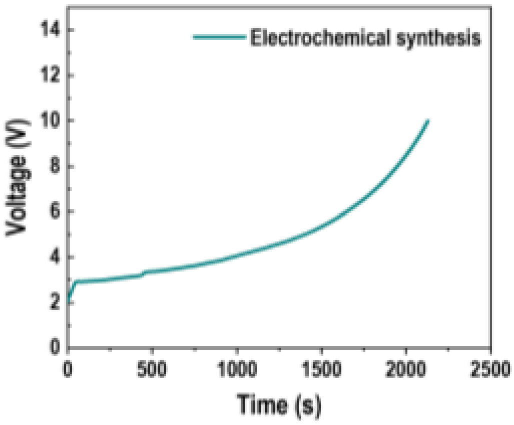

Figure S3. Voltage-time curves during fast current-driven synthesis (FCDS).

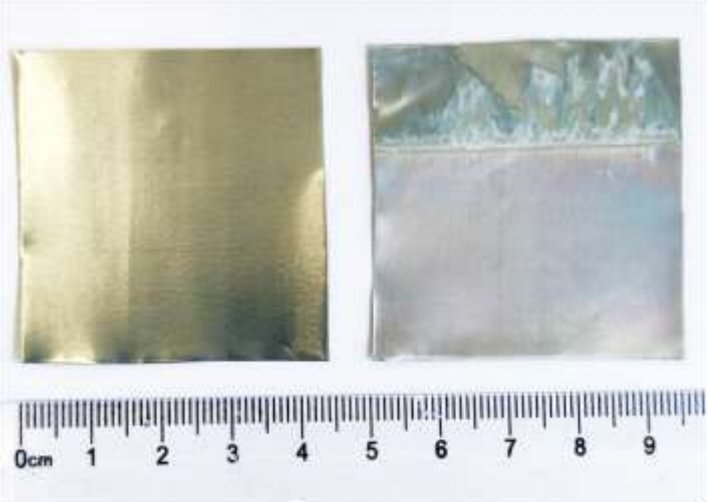

Figure S4 Photographs of the bare Zn and ZIF-7x-8 layers on Zn foil.

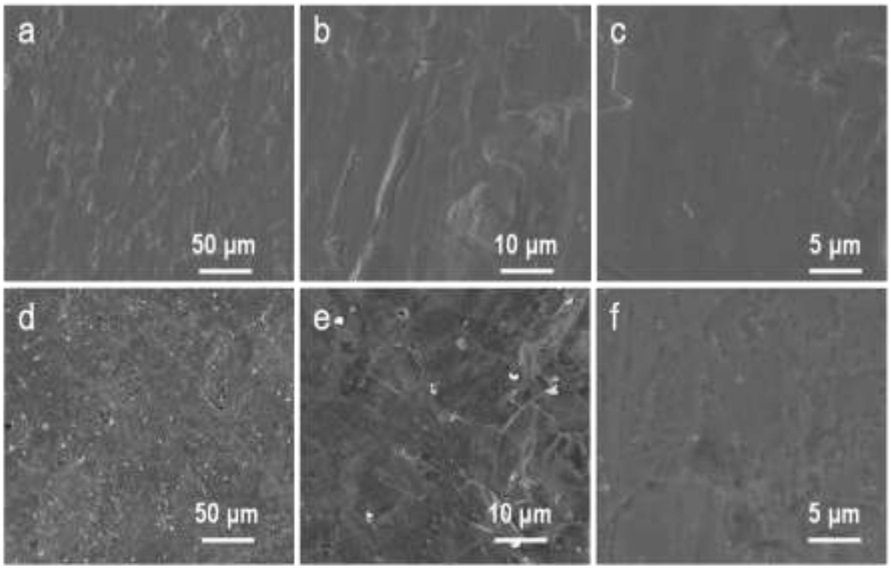

Figure S5. (a-c) SEM images of bare Zn surface. (d-f) SEM images of ZIF-7x-8@Zn metal surface.

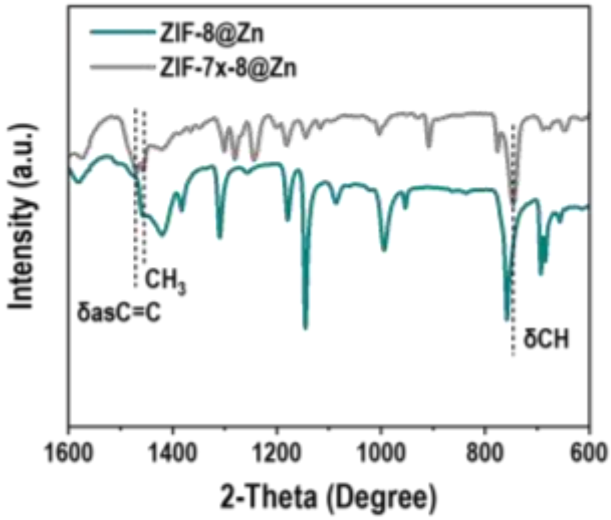

Figure S6. FT-IR spectra of the pure ZIF-8 and mixed linker ZIF-7_x-8 layer on Zn foil.

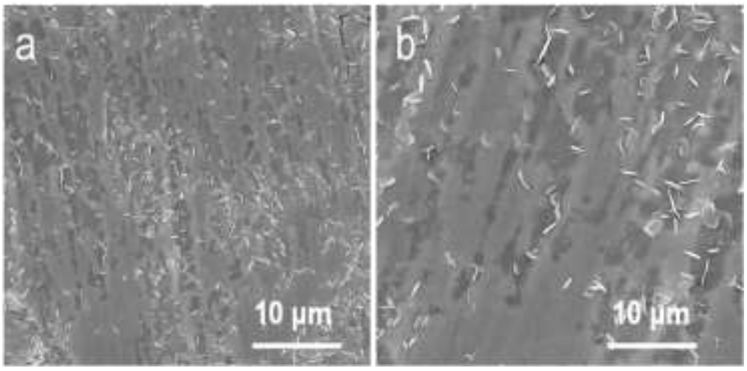

Figure S7. SEM image of the ZIF-7_x-8@Zn metal after being soaked in electrolyte for 5 days.

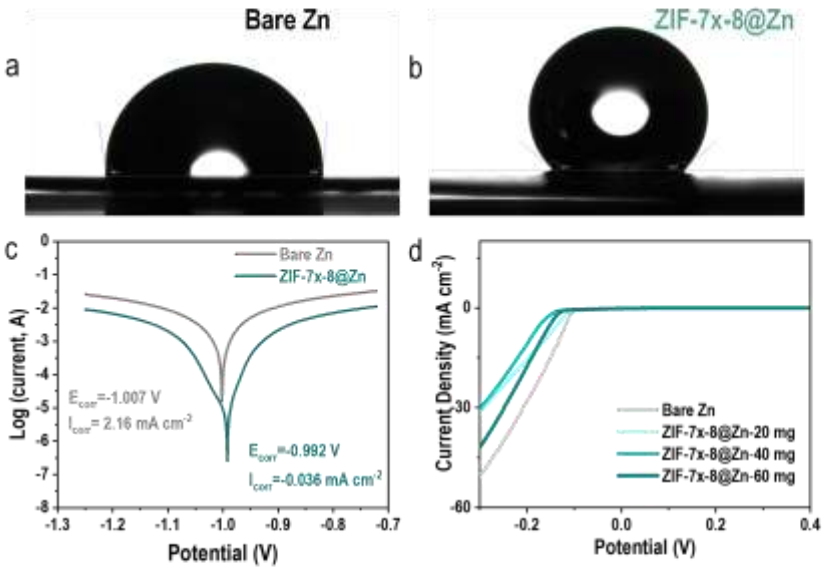

Figure S8. Contact angles of the electrolyte with a) bare Zn, b) ZIF-7_x-8@Zn anodes. (c) Corrosion curves in 2 M $ZnSO_4$-H_2O, (b) LSV curves and their corresponding Tafel plots of the bare Zn and ZIF-7_x-8@Zn with the amount of different BIM additions.

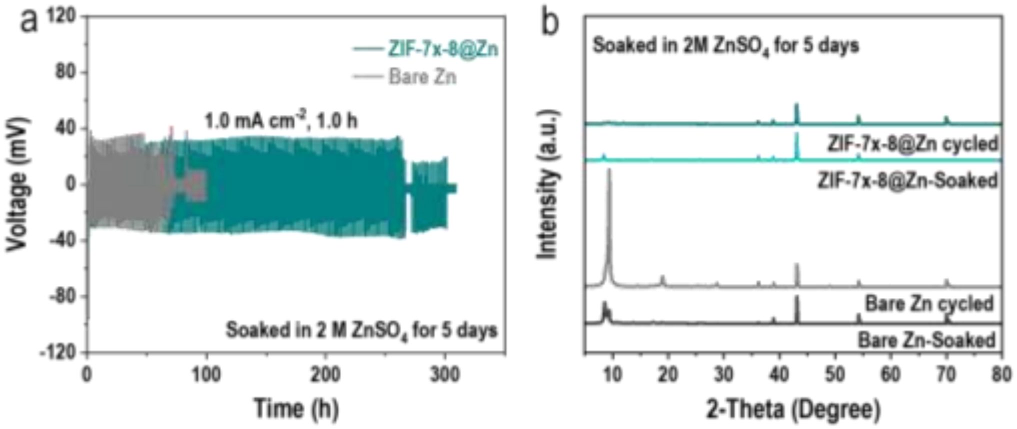

Figure S9. (a) Cycling performance of symmetric cells based on bare Zn and ZIF-7_x-8@Zn anodes which had being soaked in 2 M $ZnSO_4$-H_2O for 5 days. (b) XRD patterns of cycled bare Zn and cycled ZIF-7_x-8@Zn after being soaked.

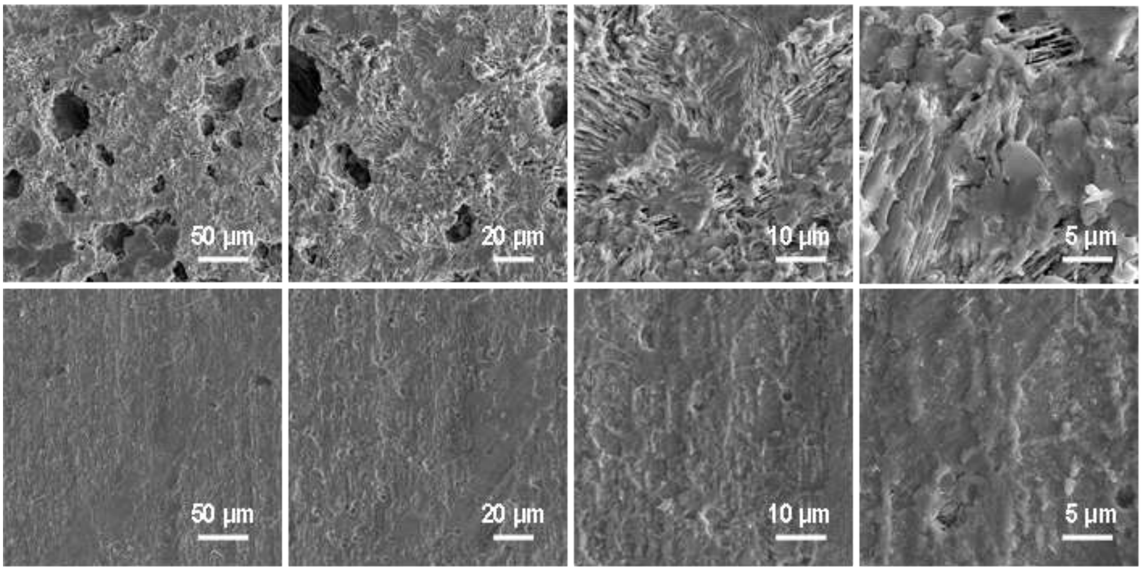

Figure S10. SEM images of (a-d) bare Zn, (e-h) ZIF-7_x-8@Zn after being immersed in 2M $ZnSO_4$-H_2O electrolyte for 5 days and circulated in symmetrical cells for 50 hours.

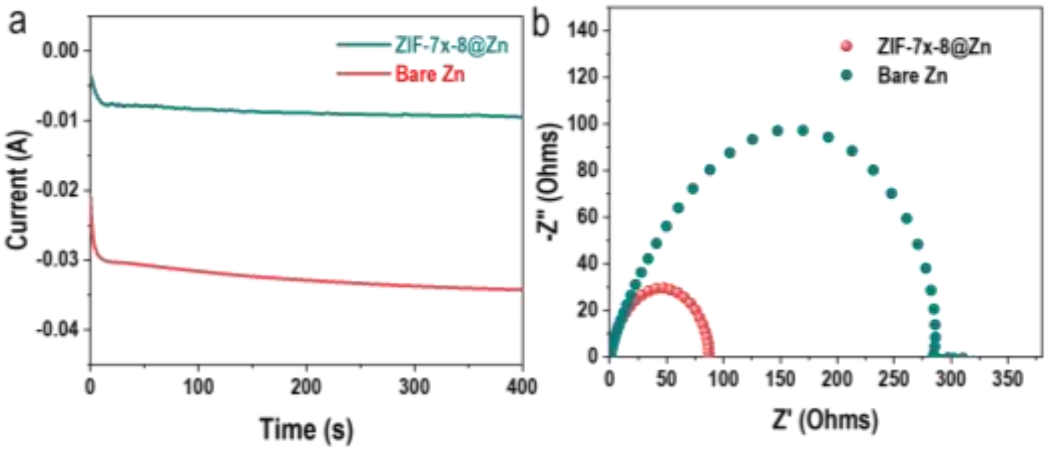

Figure S11. (a) Chronoamperograms of bare Zn and ZIF-7_x-8@Zn using a bias voltage of -200 mV. (b) EIS spectra of the symmetrical cells using bare Zn or ZIF-7_x-8@Zn as the electrode.

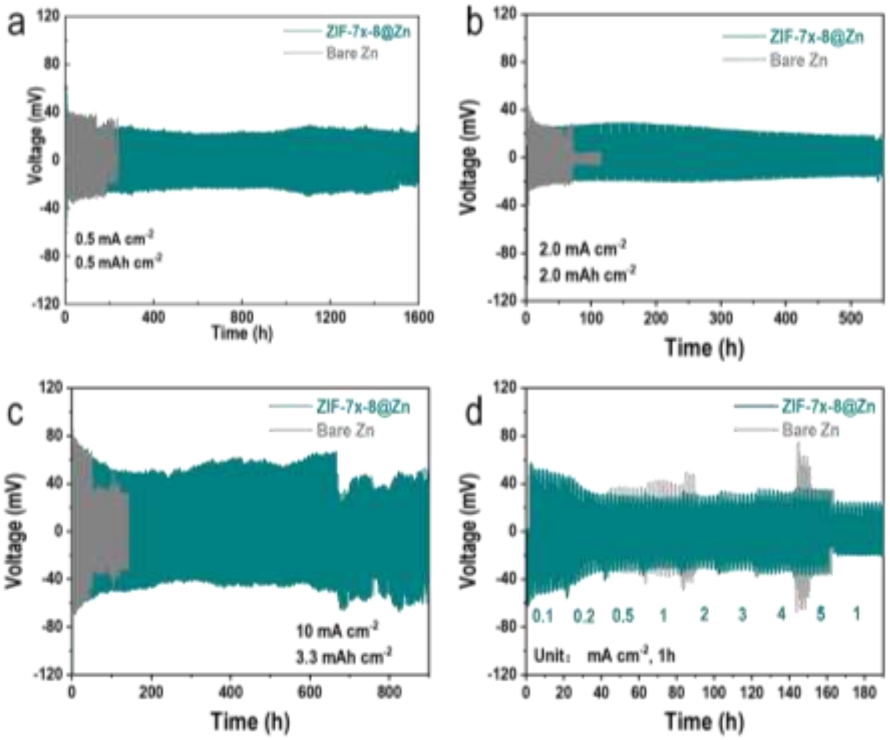

Figure S12. Electrochemical performances of Zn//Zn symmetrical cells based on both bare Zn and ZIF-7_x-8@Zn under different current and capacity conditions. (a. 0.5 mAh cm^{-2} at 0.5 mA cm^{-2}, b. 2 mAh cm^2 at 2 mA cm^{-2}, c. 10 mAh cm^{-2} at 3.3 mA cm^{-2}, d. rate performance of the bare Zn and ZIF-7_x-8@Zn electrodes, respectively).

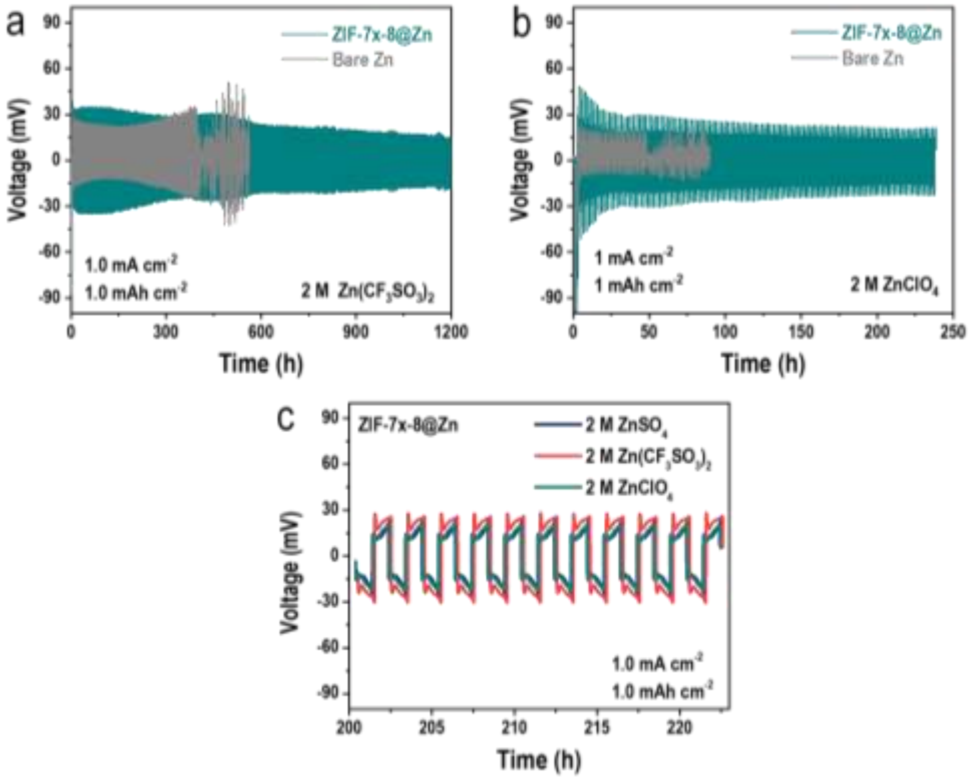

Figure S13. Cycling performance of symmetric cells based on bare Zn and ZIF-7_x-8@Zn anodes in (a) $Zn(CF_3SO_3)_2$-H_2O and (b) $ZnClO_4$-H_2O at a current density of 1.0 mA cm^{-2} and an area capacity of 1.0 mAh cm^{-2}. (c) The enlarged voltage evolution profiles of ZIF-7_x-8@Zn//ZIF-7_x-8@Zn symmetric cells in different electrolytes.

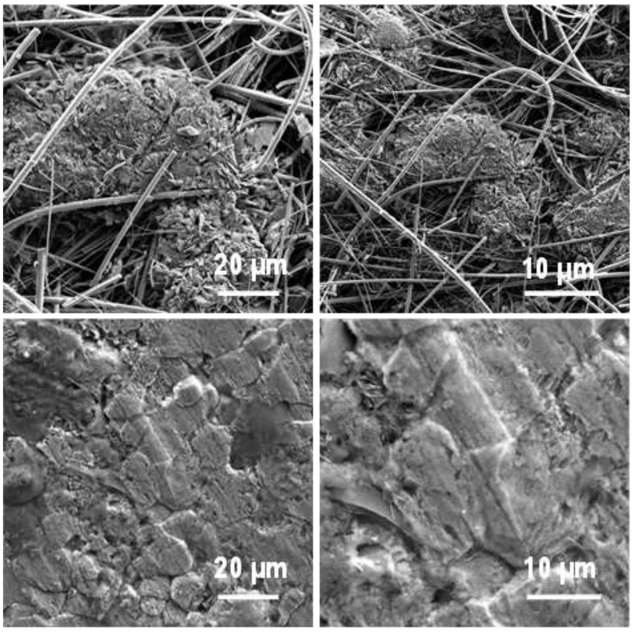

Figure S14. SEM images of (a-b) cycled bare Zn metals and (c-d) cycled ZIF-7_x-8@ Zn metals in $ZnClO_4$-H_2O under condition of 1 mAh cm^{-2} at 1 mA cm^{-2}.

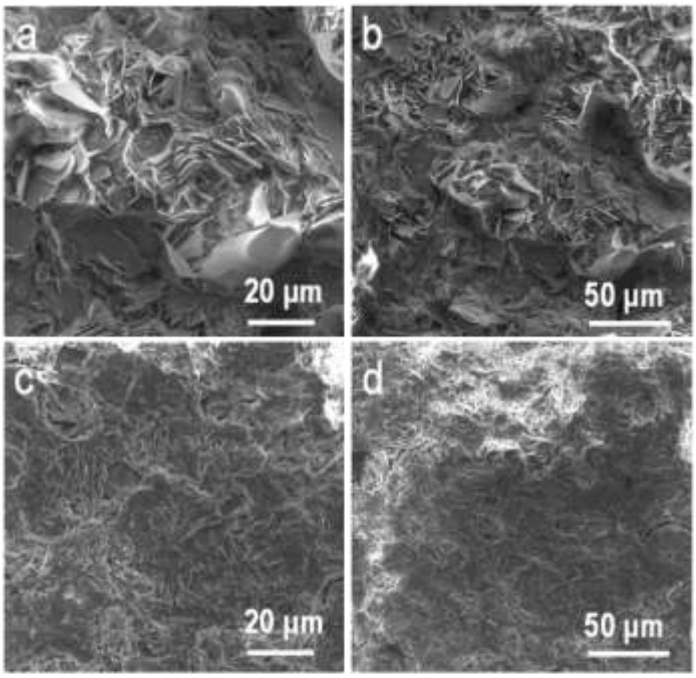

Figure S15. SEM images of (a-b) cycled bare Zn metals and (c-d) cycled ZIF-7_{x}-8@ Zn metals in $Zn(CF_3SO_3)_2$-H_2O under condition of 1 mAh cm^{-2} at 1 mA cm^{-2}.

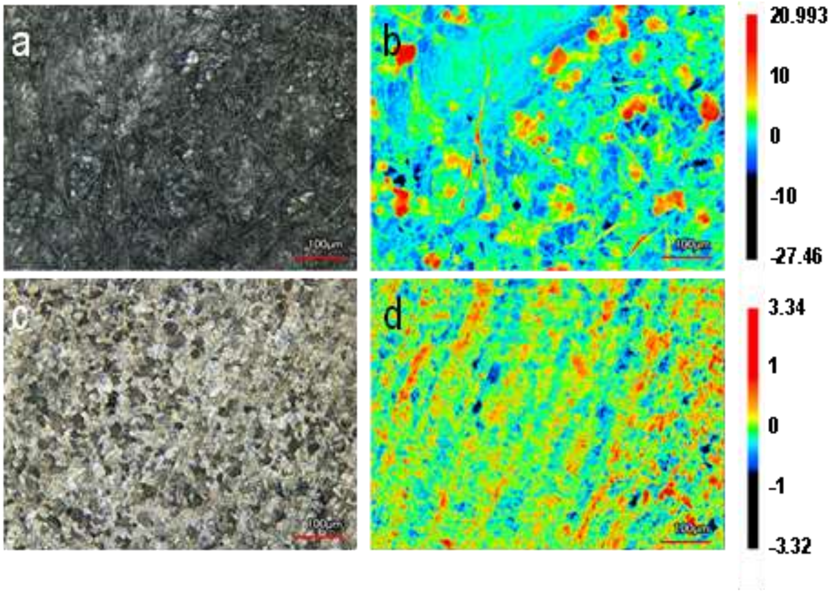

Figure S16. The Two-dimensional images and the surface height difference of the bare Zn and etched Zn after cycling.

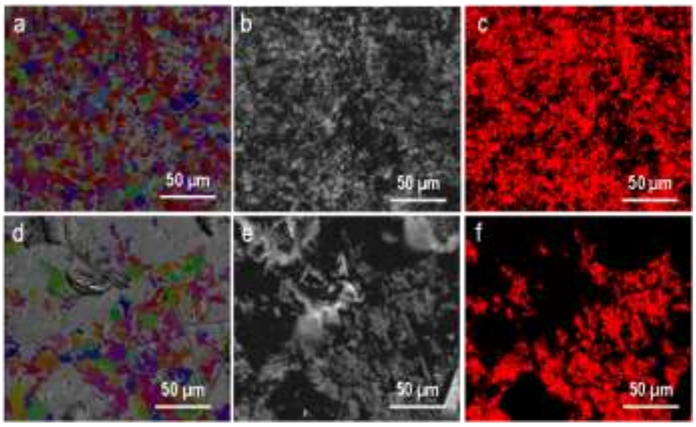

Figure S17. EBSD results of (a-c) cycled ZIF-7_x-8@Zn and (d-f) cycled bare Zn electrodes for 100 h.

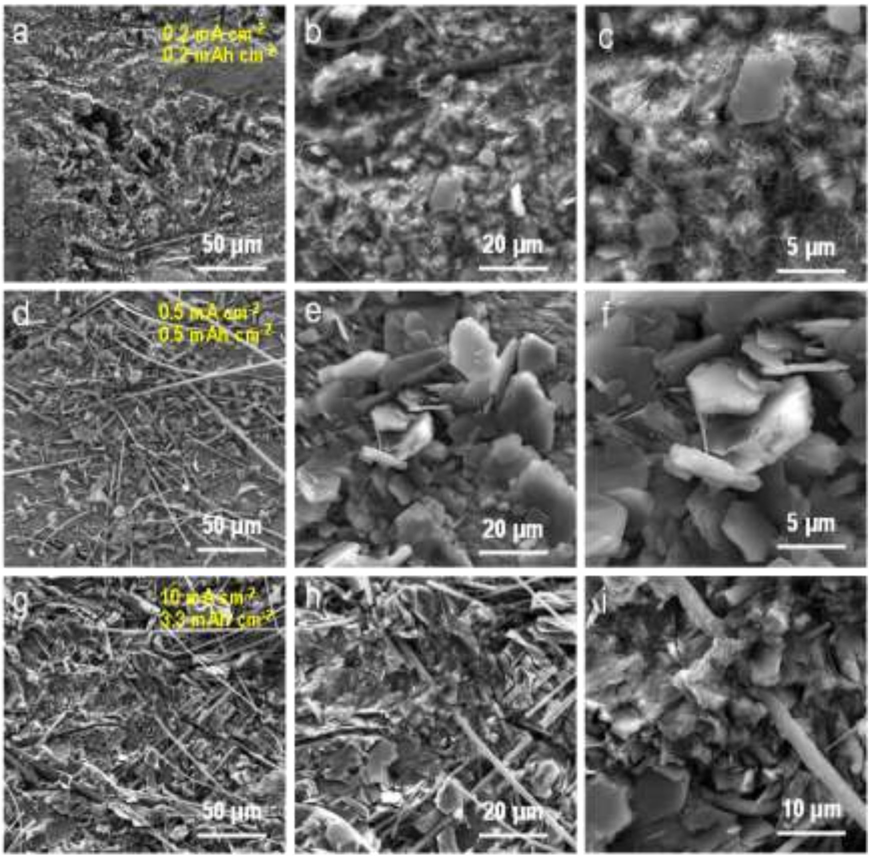

Figure S18. SEM images of cycled Bare Zn metals under condition of (a-c) 0.2 mAh cm^{-2} at 0.2 mA cm^{-2}, (d-f) 0.5 mAh cm^{-2} at 0.5 mA cm^{-2}, (g-i) 3.3 mAh cm^{-2} at 10 mA cm^{-2}.

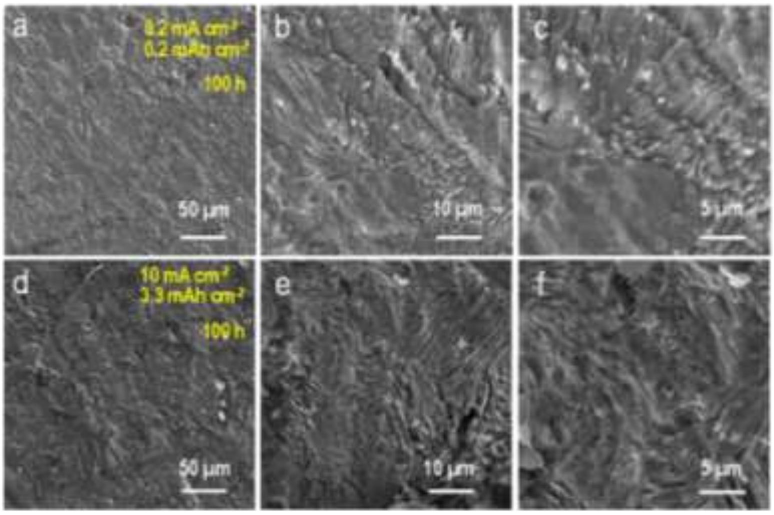

Figure S19. SEM images of cycled ZIF-7_x-8@Zn metals under condition of (a-c) 0.2 mAh cm^{-2} at 0.2 mA cm^{-2} and (d-f) 3.3 mAh cm^{-2} at 10 mA cm^{-2}.

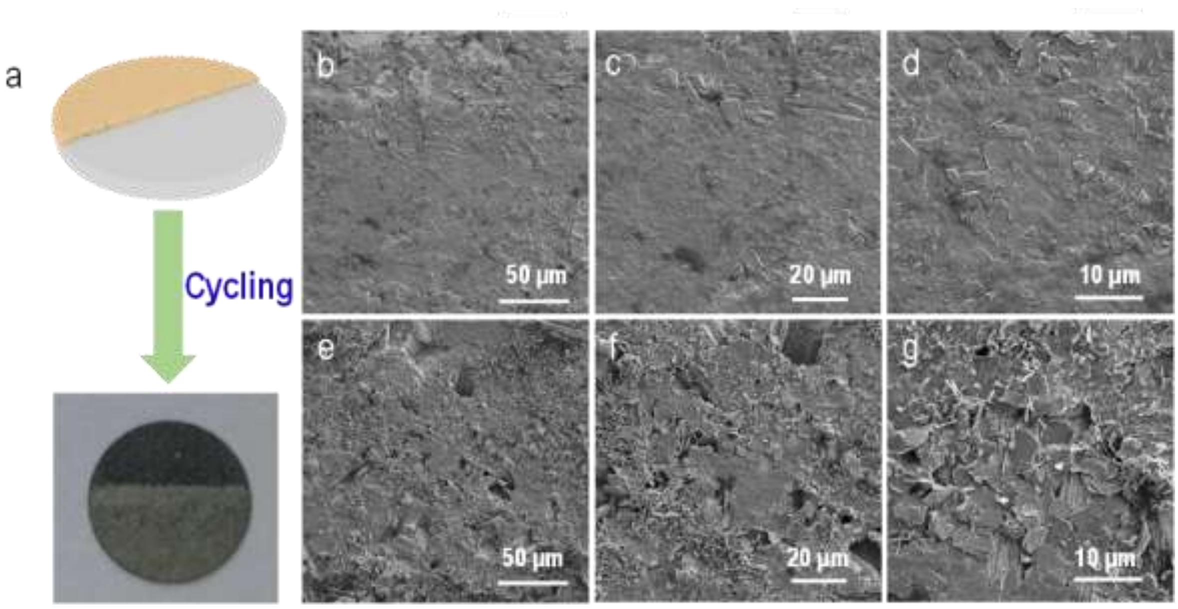

Figure S20. (a) Schematic illustration and photos of cycled metal anode which half under the protection of ZIF-7_x-8. (b-d) SEM images of district in cycled ZIF-7x-8@Zn, (e-g) SEM images of district in cycled bare Zn.

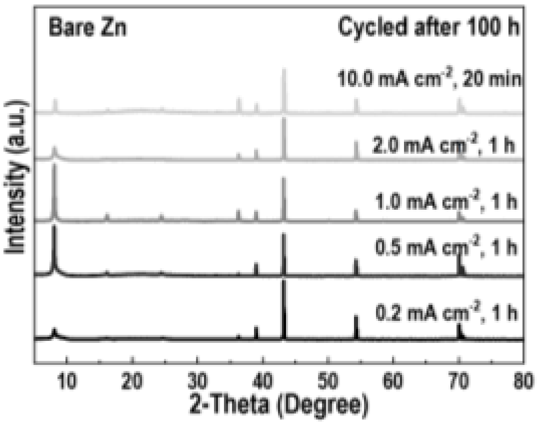

Figure S21. XRD patterns of bare Zn anodes after cycling at different current densities and area capacities.

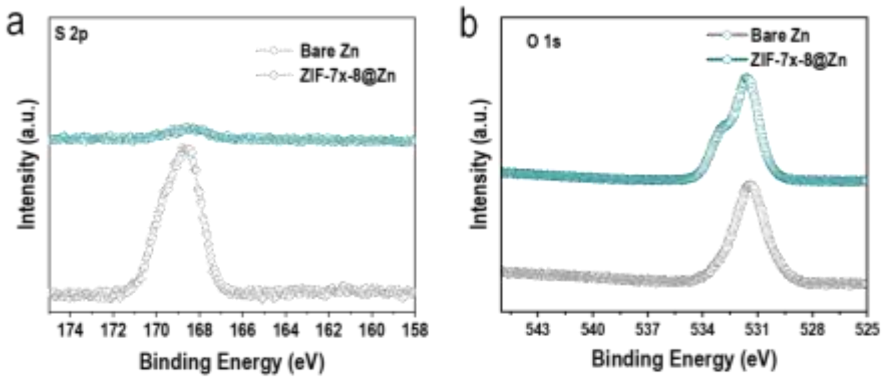

Figure S22. X-ray photoelectron spectroscopy (XPS) of cycled Zn and ZIF-7_x-8@Zn anodes.

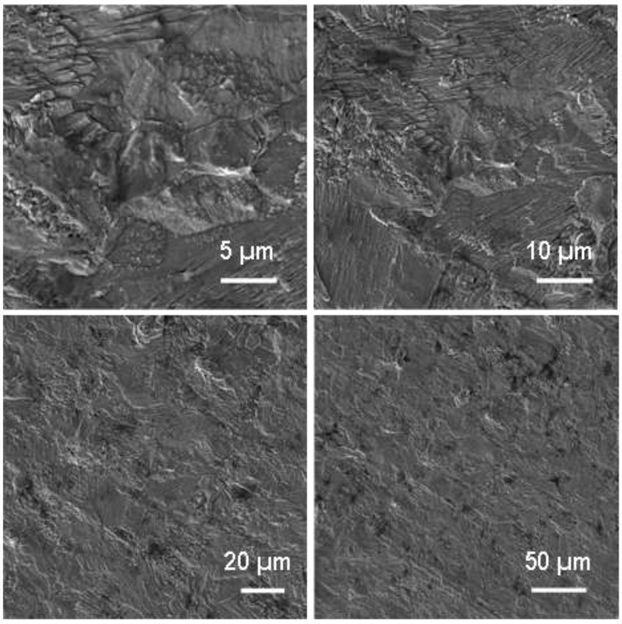

Figure S23. SEM images of Zn surface after cycling for 20 h in ZIF-7_x-8@Zn metal anode.

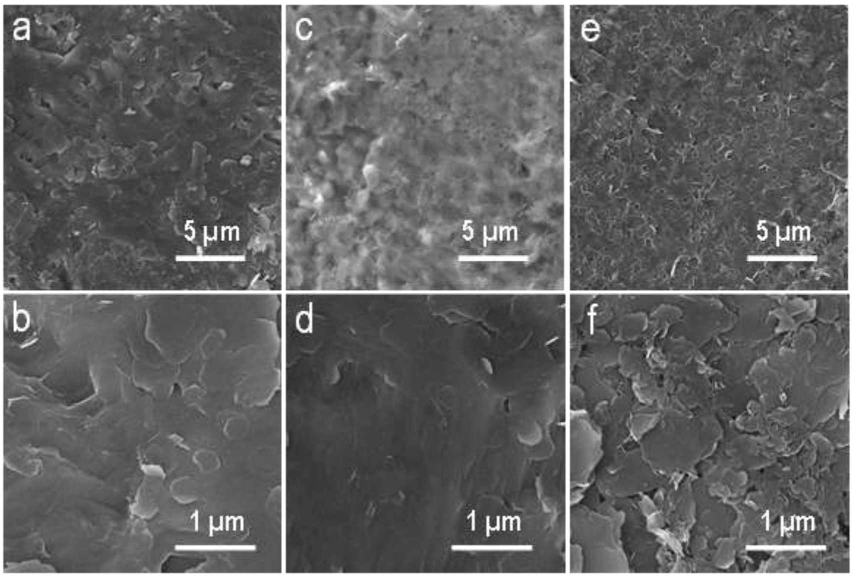

Figure S24. SEM images of Zn surface after different cycling (a-b. 150, c-d. 200, e-f. 300 hours) in ZIF-7_x-8@Zn anodes

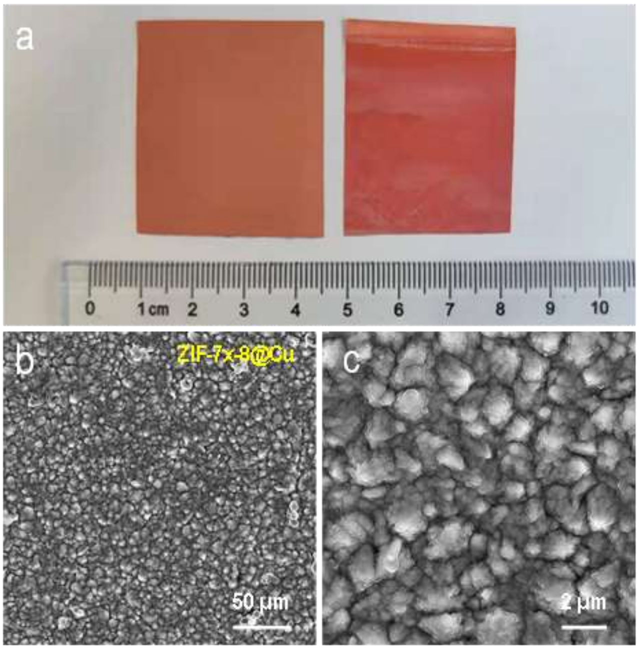

Figure S25. Photographs and SEM images of Cu surface in ZIF-7_x-8@Cu.

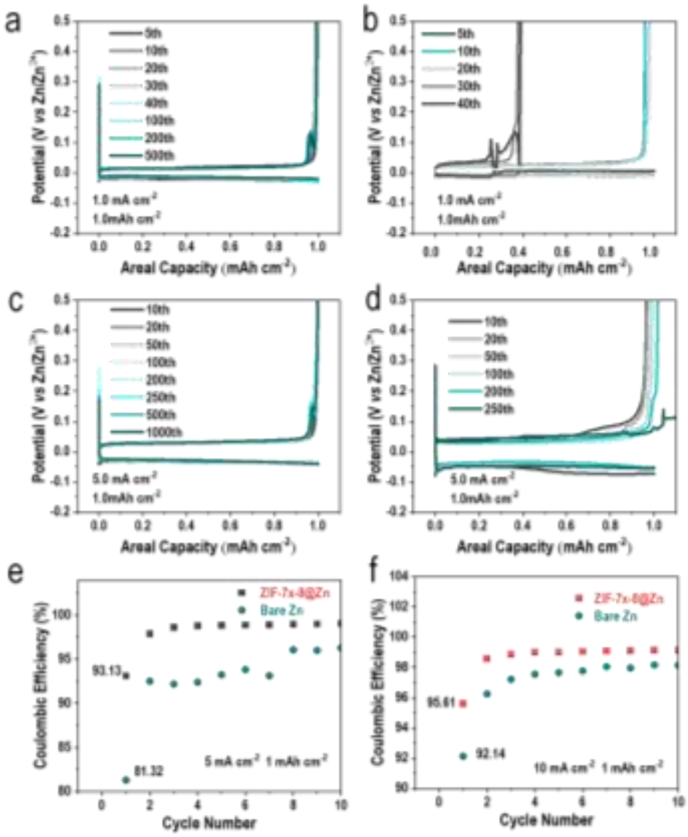

Figure S26. Voltage profiles of (a, c) ZIF-7_x-8@Zn|| ZIF-7_x-8@Cu and (b, d) Zn||Cu half-cells at different conditions (1 mAh cm^{-2} at 1 mA cm^{-2}, 1 mAh cm^{-2} at 5 mA cm^{-2}). (e, f) The CE values in the initial 10 cycles of Zn//Cu cells at 5 or 10 mA cm^{-2} with cycling capacity of 1 mAh cm^{-2}.

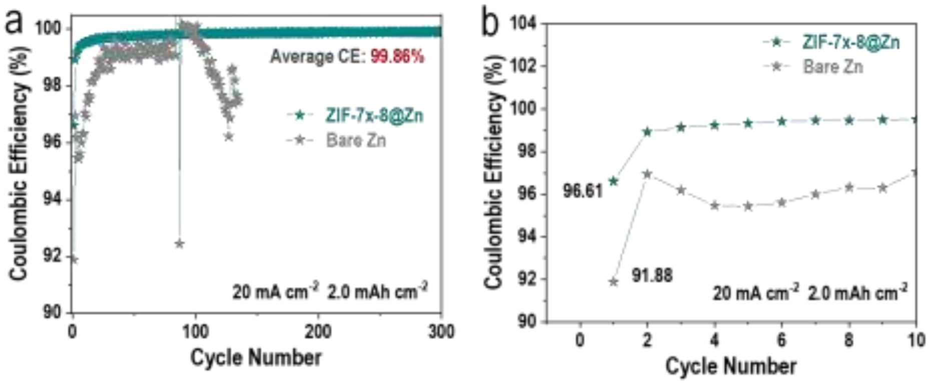

Figure S27. CEs and the initial 10 cycles of Zn//Cu half-cells based on both bare Zn and ZIF-7_x-8@Zn at 20 mA cm^{-2} with cycling capacity of 2 mAh cm^{-2}.

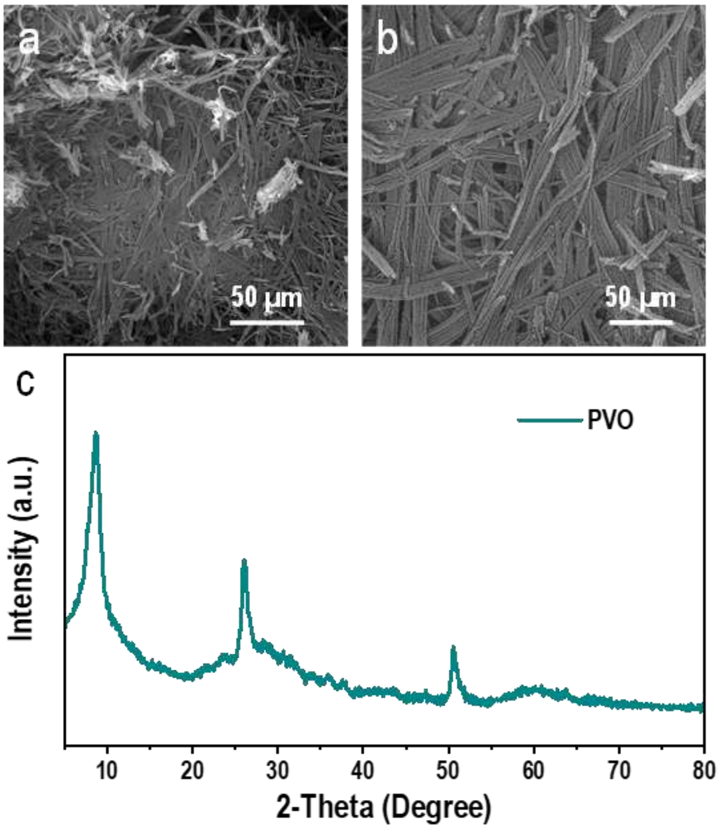

Figure S28. (a, b) SEM images and (c) XRD pattern of the synthesized PANI-V_2O_5 (PVO) cathode material.

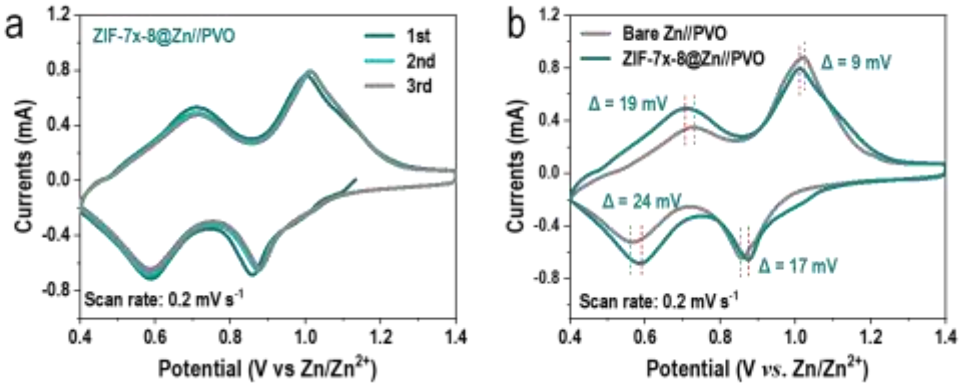

Figure S29. (a) CV profiles for the first five cycles and (b) CV curves at 0.2 mV s^{-1} of Zn/PVO batteries with bare Zn and ZIF-7_x-8@Zn anode.

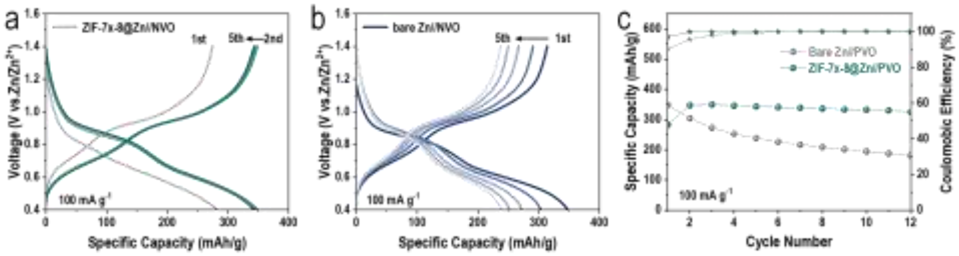

Figure S30. The voltage profile and cycling performance of the bare Zn//PVO and ZIF-7_x-8@Zn//PVO batteries at a current density of 100 mA g^{-1}.

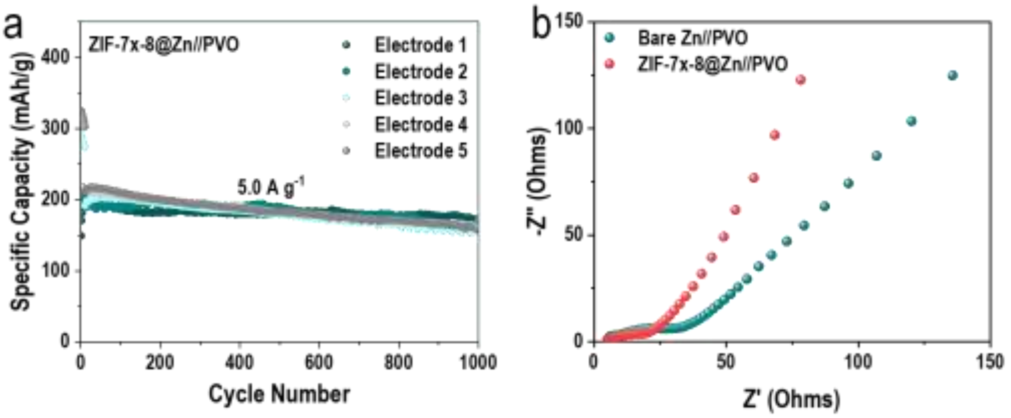

Figure S31. (a) The cycling and specific capacity test results of multiple ZIF-7_x-8-Zn//PVO cells at 5.0 A g^{-1}. (b) Electrochemical impedance spectroscopy of Zn//PVO cells based on different anodes.

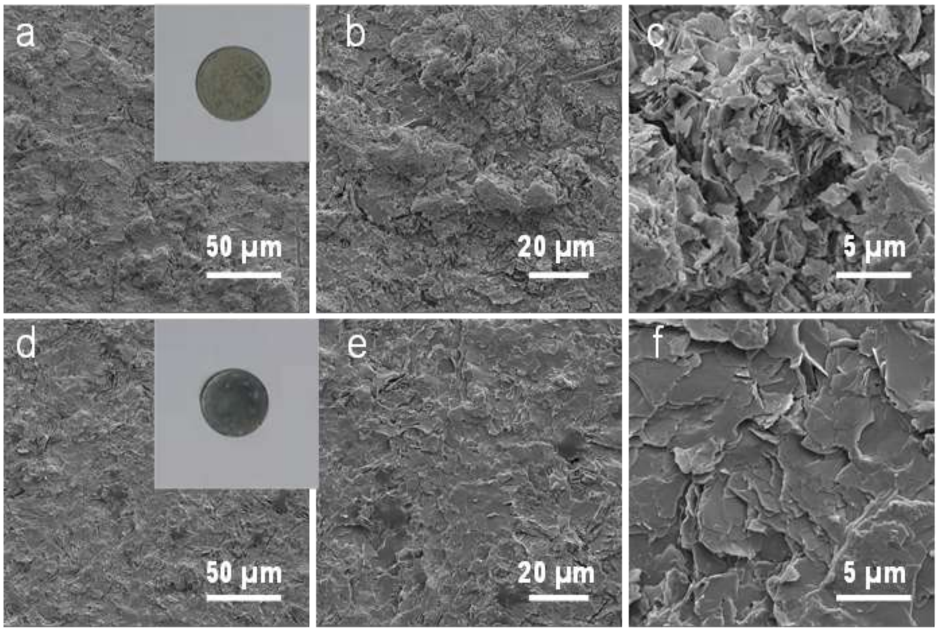

Figure S32. SEM of the bare Zn and ZIF-7_x-8@Zn anode after 1000 cycles in PVO//Zn cells at 5 A g^{-1}.

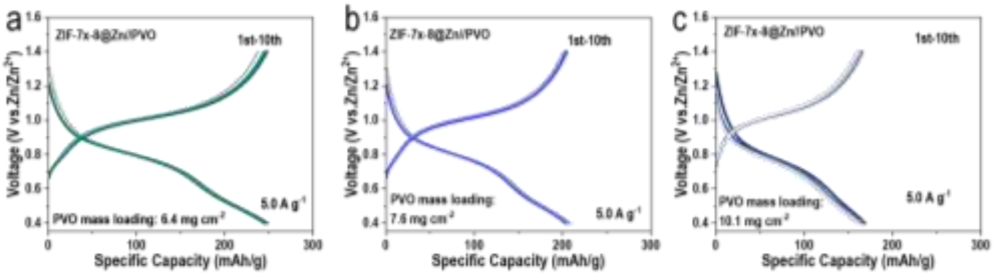

Figure S33. Voltage profile of PVO//ZIF-7_x-8@Zn batteries at current density of 5.0 A g^{-1} with different PVO mass loading after three pre-circulations.

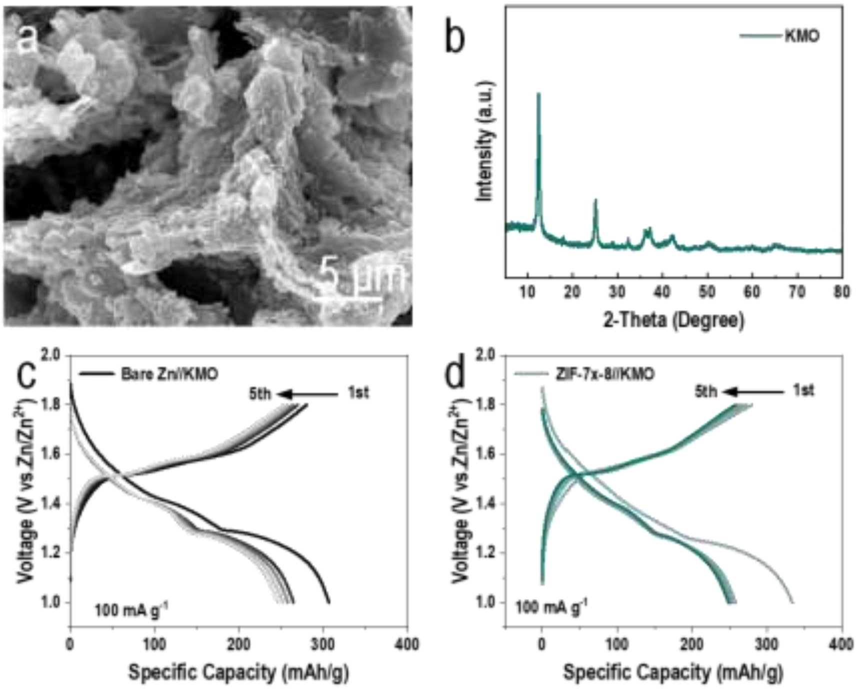

Figure S34. (a) The SEM images and (b) XRD pattern of $K_{0.27}MnO_2$ (KMO) powder. (c-d) the voltage profile of bare Zn//KMO and ZIF-7_x-8@Zn//KMO batteries at a current density of 100 mA g^{-1}.

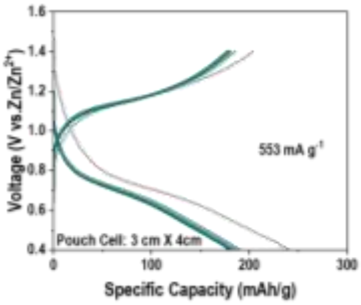

Figure S35. Voltage profile of PVO//ZIF-7_x-8@Zn pouch cell at 0.553 A g^{-1}.

Table S1. The grain indexing rate and phase percentage in EBSD of the cycled bare Zn and cycled ZIF-7_x-8@Zn.

Samples	Phase name	Percentage of phase (%)	Phase counting	Average band contrast	Minimum band contrast	Maximum band contrast
Bare Zn-cycled	Zinc	24.23	16634	84.72	14.00	205.00
	Zero resolution	75.77	52010	50.19	0.00	251.00
Etched Zn-cycled	Zinc	50.00	30243	86.57	18.00	189.00
	Zero resolution	50.00	30249	36.84	0.00	168.00
